# Mass spectrometry imaging for plant biology: a review

**DOI:** 10.1007/s11101-015-9440-2

**Published:** 2015-10-13

**Authors:** Berin A. Boughton, Dinaiz Thinagaran, Daniel Sarabia, Antony Bacic, Ute Roessner

**Affiliations:** Metabolomics Australia, School of BioSciences, The University of Melbourne, Parkville, VIC 3010 Australia; School of BioSciences, The University of Melbourne, Parkville, VIC 3010 Australia; ARC Centre of Excellence in Plant Cell Walls, School of BioSciences, University of Melbourne, Parkville, VIC 3010 Australia; Bio21 Molecular Science and Biotechnology Institute, University of Melbourne, Parkville, VIC 3010 Australia

**Keywords:** Biochemistry, Lateral resolution, Natural products, Spatial mapping, Spatial metabolomics

## Abstract

Mass spectrometry imaging (MSI) is a developing technique to measure the spatio-temporal distribution of many biomolecules in tissues. Over the preceding decade, MSI has been adopted by plant biologists and applied in a broad range of areas, including primary metabolism, natural products, plant defense, plant responses to abiotic and biotic stress, plant lipids and the developing field of spatial metabolomics. This review covers recent advances in plant-based MSI, general aspects of instrumentation, analytical approaches, sample preparation and the current trends in respective plant research.

## Introduction

A resurgence in surface-based analytical technologies and application of molecular imaging techniques is enabling new spatial and temporal exploration of metabolic processes in plant biology. Spatial analysis of plant tissues, including determination of the locations of production, storage and site/s of action of plant natural products, provides fundamental and unique insights into plant biology. A number of different molecular imaging technologies and modalities are employed, with each possessing distinct advantages and disadvantages. Mass spectrometry imaging (MSI) is gaining in popularity and is emerging as one of the leading technologies. Over the past decade MSI has been adopted for the investigation of plant biology, including mechanisms of plant responses to stresses both abiotic and biotic, plant defense mechanisms, beneficial symbiotic relationships, and fundamental ecophysiologically important processes including nitrogen fixation and nutrient cycling. Many reviewers have published excellent comprehensive reviews of MSI which have mostly focused on biomedical applications (Addie et al. 2015; Aichler and Walch 2015; Chaurand 2012; Chughtai and Heeren 2010; Ellis et al. 2014a; Gode and Volmer 2013; Jungmann and Heeren 2012; Miura et al. 2012; Norris and Caprioli 2013; Rompp and Spengler 2013; Seeley and Caprioli 2012; Shariatgorji et al. 2014; Spengler 2015; Svatoš 2010; Wu et al. 2013); more recently a number have provided coverage of imaging of plant metabolites (Aichler and Walch 2015; Bjarnholt et al. 2014; Fujimura and Miura 2014; Horn and Chapman 2014; Kaspar et al. 2011; Sparvero et al. 2012; Spengler 2015; Sumner et al. 2015). The intention of this review is to provide an introduction to MSI used for plant-based research, including an overview of the technology with a detailed review of recent developments (2013–2015), a period that has seen rapid advances. We also highlight the new technologies that have the potential to impact on “systems-based” approaches to advance knowledge of relevance to plant biology and biotechnology.

The ‘omics’ technologies, genomics, transcriptomics, proteomics and metabolomics (and others), have provided insights into plant biochemistry, physiology and biology and are at the forefront of discovery in modern Systems Biology (Sumner et al. 2015). Advanced genomics capabilities have enabled the rapid and comprehensive determination and assembly of a number of plant genomes. However, the prediction and annotation of the functions of individual genes remain notoriously difficult (Claros et al. 2012; Korte and Farlow 2013). Alongside the greater ability to identify the number of gene loci in plants, the concomitant desire to elucidate the function of these genes has led to the development of the fields of transcriptomics, proteomics and metabolomics. The products measured within the transcriptome, proteome and metabolome are all dynamic and are both spatially and temporally resolved within the organism relative to the ‘static’ genome, demonstrating a need for both spatial and temporal analytical techniques. The transcriptome represents the complement of RNA transcripts produced from the genome which varies from cell to cell or between tissue types and with development; the proteome represents the total protein complement translated from the genome which is highly localized, one gene often encoding proteins with diverse functions distinguished by the large array of post-translational modifications (PTMs) that modulate function and activity. Finally, the metabolome is comprised of the complement of small molecules or metabolites representing the end products of both anabolic and catabolic cellular processes. There is an estimated 200,000 metabolites in *Plantae*, with only 100,000 that have been isolated and identified (Fiehn 2002). Considerable work is still needed to identify the full range of natural products and the novel biosynthetic pathways employed to generate them. The metabolome is generally the first to be affected by changing conditions and measurement provides a rapid and direct determination of the phenotype or current state of an organism, providing a detailed snapshot of the complement of small molecules that can be mapped back onto metabolic pathways. The metabolome can provide more detailed information relative to individually measuring the genome, transcriptome or proteome. The distribution of metabolites and proteins within an organism is spatio-temporally resolved, and MSI offers the ability to measure both in a spatially resolved manner and at high resolution.

Plants are inherently compartmentalized into specialized groups of cells (tissues and organs) and cells into subcellular organelles/compartments, where specific biochemical processes take place, supporting life, and leading to the synthesis of a range of molecules including metabolites, phytochemicals and natural products. The biosynthesis and storage of plant metabolites are highly regulated and spatio-temporally resolved; they are endogenously expressed or produced in response to specific stimuli including both abiotic and biotic stresses. The biosynthesis of natural products occurs within sub-populations of cells from which intermediates, precursors and end-point products are either transported to their site of action or locations of storage via translocation between cells or via the vasculature through the phloem and xylem. In particular, toxic or defensive metabolites are sequestered in highly specialized compartments protecting the normal cellular processes of the plant. For example the specialized oil glands of the *Eucalyptus* species store terpene essential oils and toxic formylated phloroglucinols that act to protect the plant against herbivores and as potent antibiotics. Other specialized structures include glandular trichomes (Lamiaceae) that store essential oils or individual secretory cells, such as those found in the tissues of ginger (Zingiberaceae) and nutmeg (Myristicaceae). Plants also produce an array of signaling molecules that are generated rapidly in highly localized and transient manners or at specific time points in their life cycle.

Both spatial and temporal approaches are necessary to unveil underlying biology in higher-order systems. Spatial analysis has been conducted using a number of different techniques which can be broadly categorized into two approaches: (1) in vitro isolation and extraction of individual tissue/cell types and (2) in situ, including in vivo, analysis using an imaging approach. The suite of technologies available for in situ imaging in plants is enormously powerful and varied; taking advantage of different physical and chemical properties to provide insight into the underlying biology. Approaches such as histochemical staining and immunolabeling coupled to optical, fluorescence or electron microscopies, employed to examine underlying tissue morphology and the spatial distribution of biomolecules, are modern-day stalwarts, but are relatively limited due to targeting of select classes of biomolecules. More recently, as the capabilities of analytical instrumentation have dramatically improved, other spectral techniques have been employed, including Fourier Transform Infrared Spectroscopy (FT-IR), synchrotron X-ray fluorescence imaging (XRF) of metal distribution in plant tissues, and nuclear imaging such as Magnetic Resonance Imaging (MRI) spectroscopy (imaging water in tissues) and Positron Emission Tomography (imaging the distribution of ^11^C and ^18^F isotopes in tissues).

Modern Mass Spectrometry (MS) has seen major technical advances over the past decade that have increased the scope, applicability and adoption of the technology in a vast array of research areas (Spengler 2015). New instrumentation provides molecular specificity and high sensitivity, and has the ability to measure a broad range of analytes at high mass resolving power with high mass accuracy across wide mass ranges. MS measures individual chemicals as ions with unique mass-to-charge (*m*/*z*) ratios. When high mass resolution MS is used, the molecular formula can be identified and in tandem mass spectrometry (MS/MS) allows (generally) unambiguous identification from unique fragmentation patterns by comparison with authentic standards. Although the concept of using MS for imaging was introduced in 1962 utilizing Secondary Ion Mass Spectrometry (SIMS) (Castaing and Slodzian 1962), it was not until the mid-90’s, with the introduction of soft ionization techniques, in particular Matrix-Assisted Laser Desorption Ionization (MALDI), and application to biomedical imaging by Spengler and Kaufmann (1994) and Caprioli et al. (1997), that MSI began to be applied to the biosciences for imaging of biomolecules, including peptides and proteins. MSI has significantly advanced, providing both high lateral (spatial) and high mass resolution capabilities using a variety of different ion sources and approaches. MSI has found extensive use in molecular pathology and histology, where the technique is used to map the spatial distribution of proteins and small molecules including drugs, lipids and endogenous metabolites within tissues (Aichler and Walch 2015; Spengler 2015). MSI has been demonstrated to have a number of advantages including a label-free analysis and the simultaneous multiplex measurement of hundreds to possibly thousands of analytes in a single imaging experiment, providing rich high-density multi-dimensional data. When MSI is combined with advanced software and data analysis techniques, it allows the virtual micro-dissection and interrogation of the molecular make-up of individual tissues. Further advances in spatial resolution have placed MSI at the forefront of single-cell metabolomics (Korte et al. 2015; Thiery-Lavenant et al. 2013). The ability to monitor the metabolism of an individual specialized cell within a tissue provides unique insights into the biology of the organism and the interaction between cell types.

**Fig. 1:**
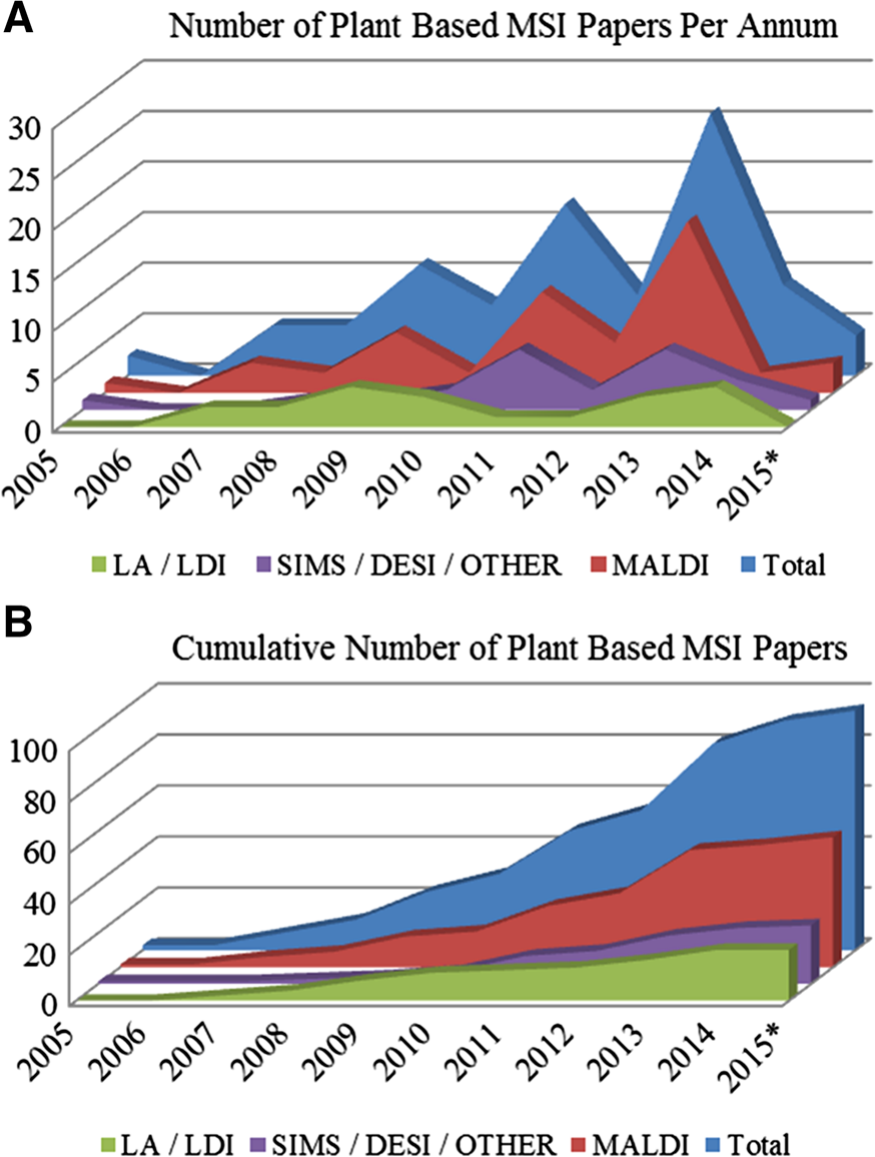
**A** Number of plant-based MSI papers per annum and **B** cumulative number of plant-based MSI papers by ionization source showing: *green* laser ablation methods (LA-ICP, LAESI) and laser desorption ionization, *purple* SIMS, DESI and other alternative ionization sources, *red* MALDI source based MSI papers, *blue* total number of papers. The cumulative number of plant-based papers by ionization source demonstrates the dominance of MALDI-type sources

For the plant biologist, MSI holds much promise for spatio-temporal analysis of plant tissues, and since 2005, the technology has been applied to measure the spatial distributions of plant metabolites allowing exploration of the functional roles of plant metabolites, including the identification of precursors or related metabolites, the exploration of localized responses to stress, and the identification of novel metabolic pathways. In comparison to mammalian tissue imaging, where the number of journal publications increased into the thousands, the number of plant-based articles totals less than 100, however, the total number of publications has been steadily increasing (Fig. 1; Table 3) (a literature search was conducted using general search terms including Imaging Mass Spectrometry, Mass Spectrometric Imaging, Mass Spectrometry Imaging and Plant Imaging, results were then filtered for plant based articles). A dramatic acceleration (doubling) in the rate of publication over the past 3 years indicates that the technology has reached a certain degree of maturity, and the approach has enough penetration and acceptance to become of utility to plant scientists.

## Basic concepts of mass spectrometry imaging

**Fig. 2:**
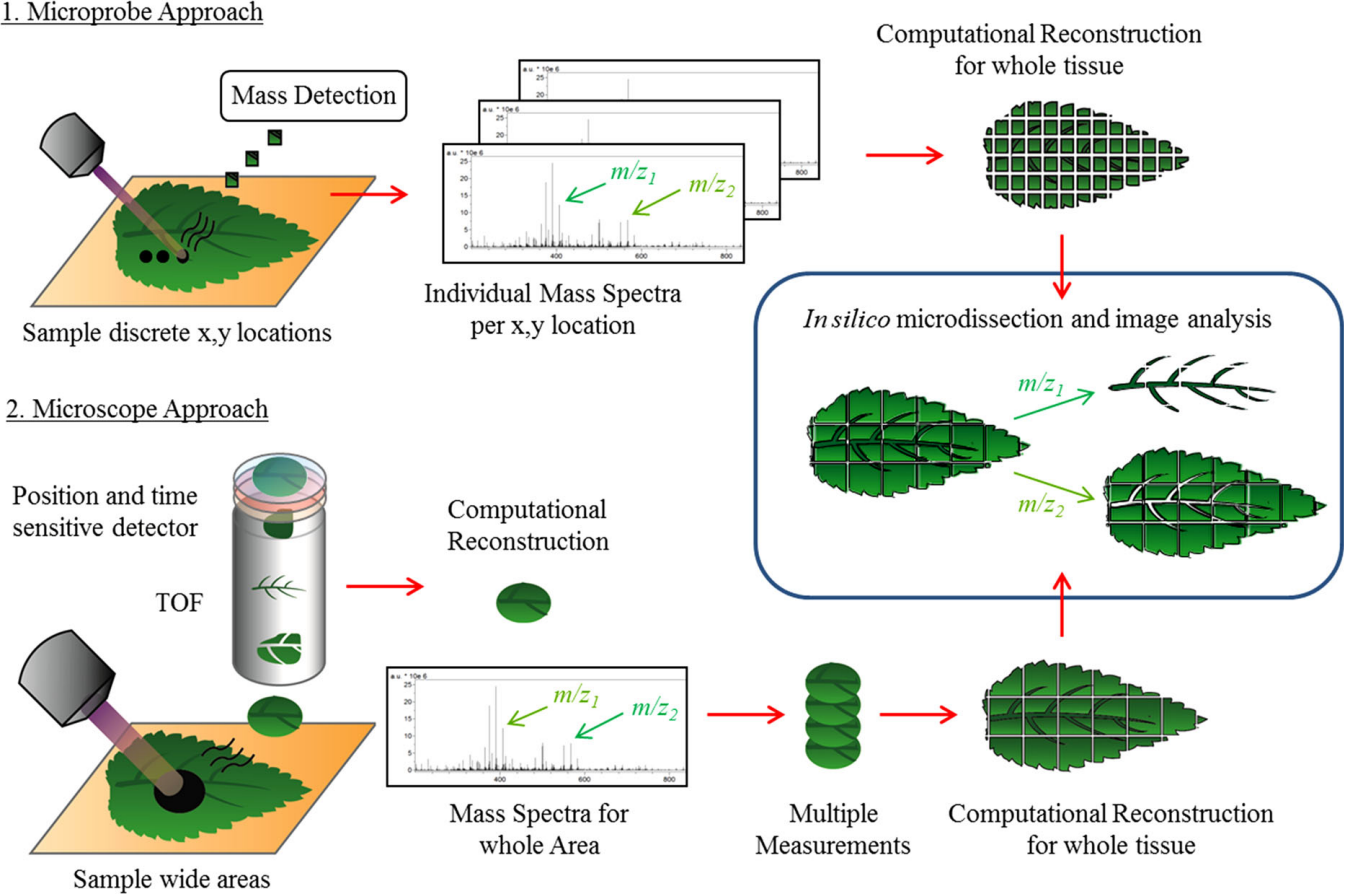
Basics of mass spectrometry imaging for MALDI ionization showing *1* microprobe approach: discrete x, y locations on tissue are sampled forming ions, the m/z of ions is measured, then resulting mass spectra for each x, y location are computationally reconstructed to form a complete dataset; *2* microscope approach: wide areas of tissue are sampled using a broadly focused laser, resulting ions are detected using a position and time sensitive mass time-of-flight (TOF) detector, allowing determination of both *m*/*z* and the discrete spatial distribution of ions within the sample area. To cover very large areas of tissue multiple measurements may be conducted across the whole tissue section with data computationally reconstructed to form a complete dataset. Image analysis is conducted in silico on datasets, individual ions may be plotted for their distribution or statistical analysis conducted to determine co-localization of ions

**Fig. 3:**
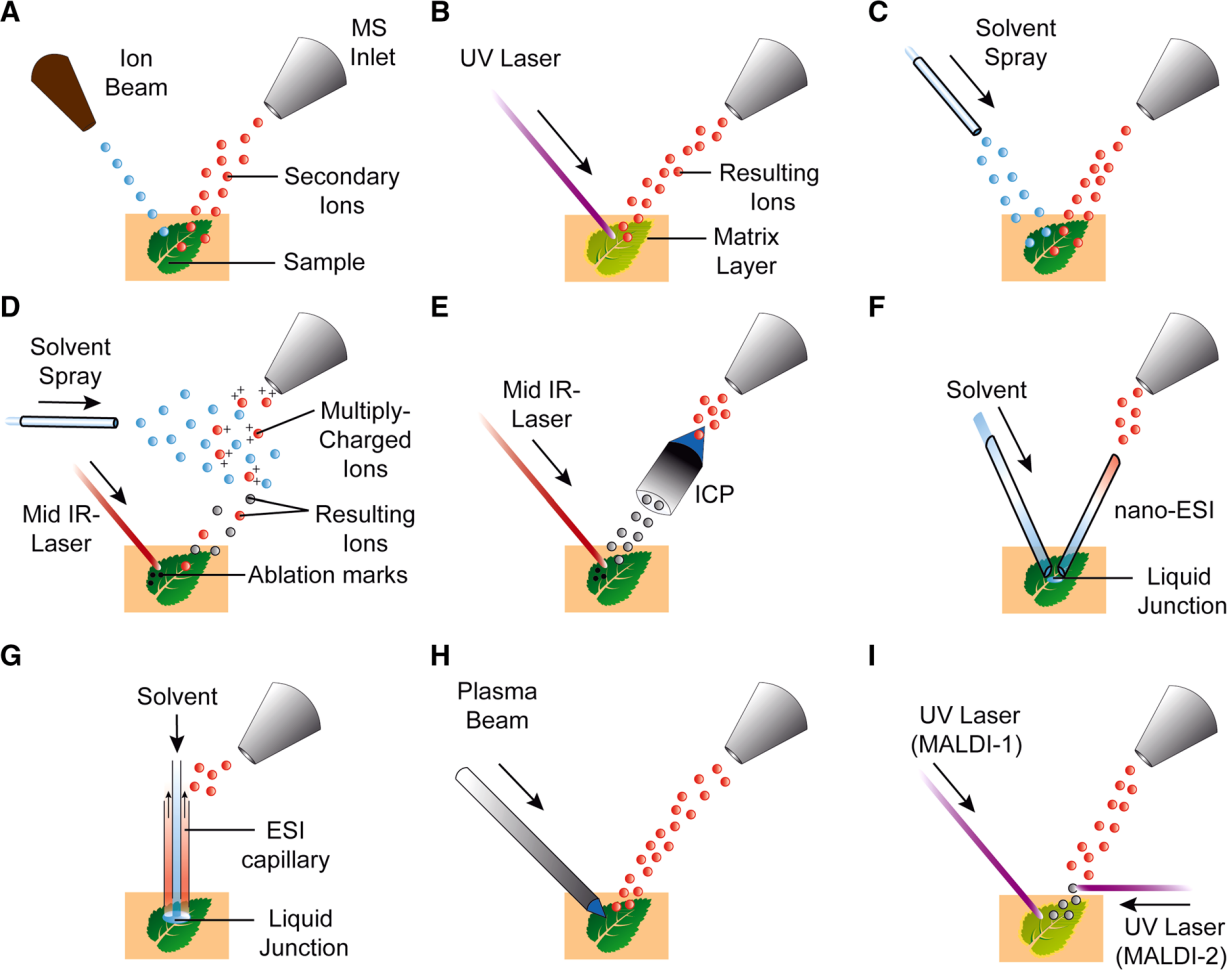
Principals of different ionization sources used for MSI imaging of plant tissues with leaf displayed, for many approaches a tissue section is used to access internal metabolites. **A** Secondary ion mass spectrometry (SIMS) showing primary ion beam impacting surface and generating secondary ions, **B** matrix assisted laser desorption ionization (MALDI) with UV laser photons absorbed by matrix layer causing desorption and ionization, **C** desorption electrospray ionization (DESI) showing electrospray stream and desorbed ions, **D** laser ablation electrospray ionization (LA-ESI) showing ablation plume and secondary ESI stream generating multiply charged ions, **E** laser ablation inductively couple plasma showing ablation (LA-ICP) plume transferred through ICP to generate ions, **F** nano-desorption electrospray ionization (nano-DESI) demonstrating micro-extraction and liquid junction followed by nano-ESI, **G** liquid extraction surface analysis (LESA) showing localized extraction and ionization through ESI capillary, **H** low temperature plasma showing plasma beam ionizing surface metabolites, **I** MALDI-2 showing primary MALDI source coupled to secondary MALDI laser inducing secondary ionization in the ablation plume. *MS* mass spectrometer, *UV* ultraviolet, *IR* infrared, *ESI* electrospray ionization, *ICP* inductively coupled plasma [modified from (Addie et al. 2015)]

A basic MSI experiment can be broken down into four steps: (1) sample selection and preparation, (2) desorption and ionization, (3) mass analysis and (4) image registration and data analysis. Careful control of each of the elements is essential to enable generation of high-quality images. In particular, sample selection, storage and preparation have a disproportionate impact on the final results; if any element in the chain is sub-optimal, then poor results will be obtained. Fundamentally, the MSI process involves placing a suitable tissue section into an ion source, ionizing the sample and collecting a series of mass spectra. This series of individual mass spectra is collected in a two-dimensional (2D) array across the tissue section or the surface of a tissue using one of a range of different ion sources and mass analyzers (Figs. 2, 3; Table 1). For each spatial co-ordinate the corresponding mass spectra collected represent the amounts of ionizable molecules present as a function of their mass-to-charge ratios (m/z). The resulting spectra are correlated with a high resolution optical image of the tissue taken either before MSI or post-MSI analysis after histochemical staining to enable identification of the cell types in the tissues. Each spectrum is assigned as an individual pixel for image generation and by plotting the intensity value of a respective ion as an intensity map across a 2D array. The resultant reconstructed ion image represents the spatial distribution of the corresponding molecule(s), which can then be compared to the optical image of the tissue. Three-dimensional (3D) approaches are also possible where serial 2D arrays from sequential tissue sections (or depth profiling) from the one tissue sample are measured and then a 3D volume is reconstructed computationally to generate a 3D ion map. Two different acquisition approaches are used to conduct an MSI experiment, either a microprobe or microscope approach (Soltwisch et al. 2014) (Fig. 1). A microprobe approach is by far the most common, where sequential individual spectra are collected and then combined into a single dataset; currently all commercial instruments operate in this manner. The microscope approach is an experimental approach in development that uses a spatial micro-channel plate detector (Timepix) which is made up of an array of individual detectors that can spatially resolve ions over a larger area in a single sampling event (Ellis et al. 2014b).

**Table 1:** List of ion sources used for (plant based) MSI including ionization technique; pressure regime: high vacuum (HV), intermediate pressure to high vacuum (IP-HV) and ambient pressure; preparation steps required for MSI, applications and achievable lateral resolutions

Method	Ionization technique	Pressure regime	Preparation steps	Applications	Lateral resolution
SIMS	Primary Ion Beam Gas Cluster	HV	Tissue section and drying. For matrix enhanced SIMS additional application of matrix	Cellular biology, lipids and lipid fragments, elements, small fragments of large biomolecules	50 nm–5 µm
MALDI	Matrix, UV Laser (Nd:YAG 355 nm, Nitrogen 337.1 nm) IR-Laser (2800–3100 nm)	IP-HV	Tissue section, drying and application of matrix	Small molecule metabolites, lipids, proteins and peptides, non-covalent complexes	UV = 10–50 µm IR = 100–200 µm
LDI	UV or IR Laser	IP-HV	Tissue section and drying	Small molecule metabolites, lipids	≥10 µm
AP-MALDI	As for UV/IR MALDI	Ambient	As for UV/IR MALDI	Small molecule metabolites, lipids	≥10 µm
DESI	ESI Stream	Ambient	None for external surfaces; section and mount for internal tissues, imprint onto PTFE	Small molecule metabolites, lipids	50–200 µm
nano-DESI	nano-Capillary coupled to nano-ESI source	Ambient	No sample pre-treatment, section if needed	Analysis of complex mixtures of soluble organic and biological molecules on substrates	20 µm
LA-ESI	Desorption using IR Laser (2800–3100 nm) coupled to ESI source	Ambient	Section if needed, mount sections on flat surface, prevent condensation by using a chamber filled with inert gas	Small molecule metabolites, lipids	300–500 µm
LA-ICP	IR Laser desorption ionization by Inductively Coupled Plasma	Ambient	Section if needed	Elemental and isotopic analysis, in situ analysis of trace elements	≥10 µm
DIOS	IR/UV Laser Desorption	Ambient	No matrix, sample deposition on spots no less than 1 mm in diameter, molecules trapped on a porous silicon surface	Small molecule metabolites, lipids,	≥20 µm
NIMS	IR/UV Laser Desorption	Ambient	Tissue section or imprint, no matrix	Small molecule metabolites, lipids, proteins and peptides	≥20 µm
LESA-MS	Liquid extraction coupled to nanoESI	Ambient	None for external surfaces, section and mount for internal tissues	Micro-liquid extraction of biomolecules	1–2 mm

The spatial resolution of an MSI experiment is a key parameter and is highly dependent on the type of ion source and sample preparation conditions employed. For 2D MSI, the term spatial resolution is commonly substituted for the more accurate term of lateral resolution, which is the ability to distinguish two different features that are located side-by-side. In practice, the lateral resolution is not generally reported or determined, and when spatial resolution is reported, it is usually used to refer to the density of pixels in the acquired 2D array that make up the resultant reconstructed image. Typically, image resolution is reported as function of the 2D array of pixels spaced at preset distances, e.g. in a 50 µm × 50 µm array. However, spatial resolution is not only a combination of the elements involved in defining 2D lateral resolution, but also incorporates depth resolution which is only relevant in 3D MSI experiments. The achievable spatial resolution of any MSI experiment is derived from a combination of different parameters including (1) the sampling area or ‘spot size’, which is a function of both the size and shape of the primary desorption event (e.g. laser pulse, primary ion beam or droplet size), (2) the step size or raster size, which is the distance between sampling events, (3) the pixel size and density of pixels which define lateral binning of MS data into digital elements, which when combined determine the lateral resolution or ability to distinguish two features. For microprobe laser- or beam-based techniques the absolute lateral resolution can be increased beyond the physical limit of the spot size by the practice of oversampling, where the area of ablation is incrementally moved in smaller steps than the overall area of the sampling spot. Lateral resolution within the microscope approach is preset and is determined by the spatial arrangement of the individual detectors of the micro-channel plate, including both size and spacing.

## Ionization techniques

MSI first relies on the ability to form ions, which are then transferred under vacuum and measured by the mass analyzer (Table 1; Fig. 3). The processes of forming ions can affect both the sensitivity and selectivity, and are dependent upon the sample type and the type of ionization source employed. The past three to 5 years have seen an explosion in different types of ion sources available, particularly specialized sources for ambient ionization conditions (Monge et al. 2013). Although not all have been explored in plant MSI, there exists much promise for their future application. Currently, in the field of plant-based MSI, the dominant ion source and approach is MALDI, due to a range of commercial instruments that display high spatial resolutions, ease of use and broad range of applicability to a variety of biological applications. A review of the literature shows that MALDI accounts for more than half of the articles published and has dominated particularly in more recent years (2014–2015; see Fig. 2). An alternative matrix-free approach using Laser Desorption Ionization (LDI) is the next-most commonly used, with roughly 10–15 % of total publications. Finally, around a third of all publications use alternative ion sources, including SIMS, Desorption Electrospray Ionization (DESI) and coupled sources including Laser Ablation Electrospray Ionization (LAESI). Of the alternative sources, DESI and SIMS have generated the most number of publications (see Fig. 2).

### Secondary ion mass spectrometry (SIMS)

SIMS was first introduced in 1962 for imaging of inorganic elements, it was not until the 1970’s that SIMS was applied to biological imaging and utilizes a high energy pulsed primary ion beam (Ga^+^, ^133^Cs^+^, Au^+^, Be^3+^, ^40^Ar^+^, C_60_^+^) or gas clusters accelerated at high energy (15–25 keV) focused onto a sample surface (Fig. 3A) (Castaing and Slodzian 1962; Galle 1970; Lefevre 1975; Morrison and Slodzian 1975). Impact of the ion beam causes emission of secondary ions at each pixel to produce spatial distribution of metabolites (Fletcher et al. 2011a; Imai et al. 2005; Saito et al. 2008, 2012; Zhou et al. 2011). Modern advances in SIMS and nano-SIMS instrumentation are allowing high lateral resolutions of <100 nm and 3D depth profiling of samples. However, SIMS has a number of drawbacks for biological MSI, including the requirement for high vacuum, essential to prevent secondary ion collision with background gases, leading to a requirement that samples and analytes must be vacuum-stable. SIMS is a harsh ionization technique that, due to the large amount of energy transferred during impact of the primary ion beam, leads to significant fragmentation of analytes and potential decreases in chemical specificity. Downstream difficulties lie in identifying unique fragment ions for individual analytes from complex biological matrices where similar fragments may be observed from related or similar molecules.

### Matrix-assisted laser desorption ionization (MALDI)

MALDI is the most common MSI technique and is a soft ionization technique that enables direct measurement of molecular ions (Fig. 3B) (Caprioli et al. 1997). MALDI relies upon a secondary matrix to absorb the relatively harsh laser ablation energy. In most cases the matrix is a small organic chemical that aids both desorption of analytes from the solid to gas phase and promotes ionization within the ablation plume (Dreisewerd 2003). Using this approach, little to no fragmentation of individual analytes is observed. Depending upon the instrument configuration, MALDI instruments are capable of measuring large mass ranges, >100 kDa, enabling measurement of a broad range of biological molecules. MALDI may use a number of different laser sources, either in the Ultraviolet (UV) or Infrared (IR) range, with differing wavelengths including Nitrogen UV laser (337.1 nm wavelength), Neodynium-doped yttrium aluminium garnet UV laser (Nd:YAG, 355 nm wavelength) and tunable infrared lasers to promote desorption (Park and Murray 2012). Laser Desorption Ionization (LDI) is a matrix-free technique that relies upon volatilization of molecules by direct absorption of laser energy. This represents a more energetic approach, relative to MALDI, that can lead to significant in-source ion fragmentation of chemicals present. Ion yields for LDI are estimated in the range of 1 % of MALDI, and sensitivity can be impacted due to lower yields (Hölscher et al. 2014; Holscher et al. 2009; Thiery-Lavenant et al. 2013).

### Ambient ionization techniques

Ambient ionization techniques are growing in popularity due to the ability to measure directly off sample surfaces with very little preparation. Atmospheric Pressure Matrix-Assisted Laser Desorption Ionization (AP-MALDI) or AP High Resolution Scanning Microprobe—MALDI (AP-SMALDI) are ambient-pressure variants of the MALDI technique with high lateral resolution (spot sizes 12 μm). They typically use a nitrogen laser at AP to enable desorption and ionization (Koestler et al. 2008). The higher source pressures decrease in-source fragmentation of analytes due to collisional cooling with neutral gases within the source. The AP source also imparts a number of other advantages (relative to MALDI), including allowing direct mounting of samples, preventing vaporization and sublimation of volatile matrices and analytes within the source over time, thereby allowing measurement of samples for a longer period and resulting in images with larger pixel number and density.

### Desorption electrospray ionization (DESI)

The DESI method directs charged droplets to the surface of samples via a spray capillary, the ESI stream impacts the surface extracting and ionizing analytes (Fig. 3C) (Ifa et al. 2007; Wiseman et al. 2006; Zoltán et al. 2004). The ions are desorbed into the gas phase and then transferred via an atmospheric ion transfer line into the MS, thereby enabling measurement of ions. The sampling area of a DESI source is large, with lateral resolutions of 250 µm achievable under standard conditions. With great care, lateral resolutions of 50 µm have been achieved. A variant of DESI uses imprinting of sample analytes onto a surface, such as a PTFE membrane, paper or Thin Layer Chromatography plate, by directly pressing the sample onto the surface, thus transferring analytes which can then be imaged using a normal DESI approach (Muller et al. 2011; Thunig et al. 2011). Nanospray-Desorption Electrospray Ionization (nano-DESI) MSI utilizes a self-aspirating nanospray capillary that is translocated across a sample surface, with lateral resolutions of 100–150 µm achievable (Fig. 3F) (Lanekoff et al. 2013; Laskin et al. 2012b). The arrangement directly transports desorbed surface analytes to a nanospray-ESI source, where ionization occurs. This arrangement prevents the simultaneous desorption and ionization event and leads to improved sampling efficiency (Roach et al. 2010). Both DESI and nano-DESI do not require intensive sample preparation steps nor require high-vacuum conditions for ionization, allowing direct spatial analysis of plant surfaces (Roach et al. 2010; Zoltán et al. 2004).

### Laser ablation techniques

Laser Ablation techniques also operate under AP, including Laser Ablation Electrospray Ionization (LAESI) and Laser Ablation Inductively Coupled Plasma (LA-ICP) ionization. LAESI couples mid-infrared laser ablation of a sample surface, to generate a plume of predominantly neutral particles and molecules, to electrospray ionization (ESI) where charged droplets from the ESI ionize gas within the ablation plume (Fig. 3D) (Nemes and Vertes 2007). The ionization technique allows the generation of multiply charged ions, which can offer a number of analytical advantages for protein and peptide MSI. LAESI had previously been used for lateral imaging (300–350 µm resolution) and depth profiling (30–40 µm resolution) in plants; combination of these provides insight of 3D imaging (Nemes et al. 2008; 2009; Nemes and Vertes 2007). LA-ICP-MS is frequently used for 2D and 3D imaging of elements and isotopes in biological samples (Fig. 3E) (Becker 2013). The sample surface is ablated using a UV laser producing an ablation plume, which is then passed through inductively coupled argon plasma (ICP) at 8000 K, generating elemental ions for MS analysis. LA-ICP-MS is highly sensitive and capable of detecting elements and isotopes of low concentrations (mg/g to ng/g range) without any sample preparation (Sussulini et al. 2013).

### Desorption ionization on silica (DIOS) and nanostructure-initiator MS (NIMS)

Desorption Ionization On Silicon (DIOS) and Nanostructure-Initiator Mass Spectrometry (NIMS) use a silicon substrate to which sample analytes have been deposited or transferred directly by pressing against the surface of the sample. NIMS uses customized porous silicon surfaces (10–20 nm pores) with trapped nanostructure initiators in the pores which aid desorption and ionization (Fig. 8) (Woo et al. 2008). Laser irradiation desorbs analytes for MS analyses (Woo et al. 2008). For NIMS, the initiators are not ionized during the desorption process, leading to an improved signal-to-noise ratio with decreased interference when analyzing low-mass metabolites (in comparison to MALDI). A similar method, Nanostructured Laser Desorption Ionization (NALDI), uses a metal target coated with either nanostructures or nanowires which is pressed against a sample surface, transferring analytes to the target (Tata et al. 2014). A matrix-free laser desorption approach is then used, with desorption and ionization promoted by the nanowires.

### Liquid extraction surface analysis MS (LESA-MS)

LESA-MS is a combination of solid sample surface micro-liquid extraction and nano-electrospray MS (Fig. 3G) (Eikel et al. 2011; Kertesz and Van Berkel 2010; Tomlinson et al. 2014). Extraction solvents (acetonitrile, methanol, water:formic acid) are dispensed onto the surface of a sample extracting localized analytes, a liquid micro-junction is maintained allowing aspiration and subsequent nanospray-ionization (Kertesz and Van Berkel 2010). Although spatial resolution is poor (in the mm range), due to the large area covered by the solvent, this approach offers the ability to extract a broad range of analytes and, when coupled to separation using nano-LC–ESI–MS, offers much potential to conduct highly localized orthogonal separations, which will increase both the sensitivity and the depth of coverage.

### Other ionization sources

Instruments are frequently being ‘mixed and matched’ to produce either hybrid or multimodal configurations, improving spatial resolution and an ability to measure a wider range of compounds in the process. Recently, the dual MALDI/ESI source of a Bruker SolariX instrument was coupled to a LESA source, resulting in multimodal MALDI and LESA analysis being conducted on the same tissues (Tomlinson et al. 2014). Shimadzu have commercialized a dual optical microscope and MALDI-TOF, enabling multimodal optical and MSI imaging within the same instrument. A Laser Ablation Atmospheric Pressure Chemical Ionization (LA-APCI) multimodal optical and MSI imaging instrument was recently reported that consists of a commercial laser micro-dissection system used to isolate individual cells, which had been coupled to a modified APCI source for secondary ionization of ablated material (Lorenz et al. 2013). This hybrid instrument was capable of providing high lateral resolution (13 µm). There has also been a MALDI/SIMS hybrid MS developed by mounting a 20 kV C_60_ ion gun onto an existing MALDI ion source that was able to achieve a lateral resolution of 10 μm in mammalian neurons (Lanni et al. 2014). Very recently, a number of new sources were reported that have been optimized for MSI in plants, including a Low-Temperature Plasma probe (Fig. 3H) (Maldonado-Torres et al. 2014) and a hybrid MALDI-2 source (Fig. 3I) (Soltwisch et al. 2015), which incorporates a second wavelength-tunable post-ionization laser that initiates secondary ionization in the primary ablation plume, which has been shown to increase ionization events by several orders of magnitude over standard MALDI sources.

## Mass analysis

The mass analyzer is the core component of a MS, enabling determination of *m*/*z* of an ion. The type of mass analyzer used also has a direct impact on the ability to conduct MSI experiments (Table 2). There are three common mass analyzers used on MSI instruments, (1) unit resolution analyzers including quadrupole and ion-trap technologies, typically a linear ion trap; high resolution analyzers including, (2) Time-of-Flight (TOF) and (3) Fourier Transform (FT) encompassing both Orbitrap and FT Ion Cyclotron Resonance (FT-ICR) instruments. For metabolites there is clearly a need for accurate-mass and high mass-resolving instruments and/or the use of tandem mass spectrometry (MS/MS) to be able to distinguish different metabolites in tissues when conducting MSI experiments. Low mass-resolution instruments can lead to misidentification or misinterpretation due to inability to resolve nominally isobaric peaks (peaks very close in mass that cannot be distinguished in the acquired mass spectrum). Hybrid instruments that combine one or more different mass analyzers offer many advantages; typically a mass selective quadrupole coupled to a collision cell will be operated with a higher mass resolution analyzer such as a TOF or FT. Common arrangements include quadrupole ion-trap-TOF, Qq-TOF, Qq-FT-ICR and Q-Orbitrap systems. A number of instruments incorporate Linear Ion Traps (LIT), imparting a number of advantages including increased sensitivity due to ability to trap specific ions and increase the population of selected ions.

**Table 2:** List of common mass analyzers and instrument configurations detailing: mass resolving power, approximate mass range, tandem MS/MS capabilities and acquisition speed

Mass analyzer/configuration	Mass resolving power	*m*/*z* range	MS/MS	Acquisition speed
Ion Trap	~1000	50–4000	Yes	Medium
TOF	2500–40,000	20–500,000	No	Fast
TOF/TOF	>20,000	20–500,000	Yes	Fast
IT-TOF	10,000	50–20,000	Yes	Fast
IT-Orbitrap	>100,000	40–4000	Yes	Slow
Q-Orbitrap	>100,000	50–6000	Yes	Medium
FT-ICR	>200,000	10–10,000	Yes	Slow
Ion Mobility Q-TOF	13,000/40,000	Up to 40,000	Yes	Fast

*TOF* time of flight, *TOF/TOF* tandem TOF, *IT* ion trap, *FT-ICR* Fourier transform ion cyclotron resonance, *Q-TOF* quadrupole time of flight

Spectral resolution as a function of both sensitivity in detection and the ability to resolve different ions from each other, of the acquired mass spectra, is dependent upon both the type of mass analyzer and detector used. High-sensitivity detectors now allow the detection and amplification of very small numbers of ions, even a single ion, thus allowing measurement across wide concentration ranges, increasing the total possible number of ions observed. High mass-resolution instruments are required to distinguish very small mass differences, also increasing the total possible number of ions observed. The ability of a MS to distinguish one mass peak from an ion close in mass is described by both mass resolution and resolving power (RP). Mass resolution is defined as the degree of separation between two adjacent ions observed in the mass spectrum (*Δm*) at Full Width Half Mass (FWHM) of the peak. Resolving power is the inverse of mass resolution and is defined as the nominal mass (*m*) divided by the difference in masses (*Δm*). Higher mass-resolution allows easier identification of contributing ions to the mass spectrum, and higher mass-resolving power is essential for high mass-accuracy, whereby a higher RP allows more accurate identification of center-of-peak and determination of mass error. Low mass error is essential for unambiguous assignment of molecular formula, aiding in identification. Mass error is defined as the difference between the observed and theoretical mass of a given ion. For modern high-resolution mass spectrometers, <10 parts per million (ppm) mass error is common for TOF instruments, and <2 ppm mass error is common for FT instruments (FT-ICR and Orbitraps). The high mass-resolution available from FT instruments provides further advantages for MALDI-MSI, allowing resolution of interferences from matrix signals in the low mass-range.

Measurements conducted on low mass resolution instruments are typically operated in a targeted tandem MS approach to provide molecular selectivity, where specific fragment ions of a single analytes are monitored thereby providing both molecular specificity and increased sensitivity. For MSI measurements, higher-resolution detectors provide the ability to unambiguously resolve a peak from the complex spectra, which are generated with profiling-type techniques. Accurate mass instrumentation provides the ability to conduct profiling-type, untargeted measurements where high-resolution analyzers can distinguish nominally isobaric peaks. There are three different types of high-resolution analyzers in common use, including high mass resolution TOF with achievable resolution up to 60,000 (typically 10–50,000), ultra-high mass resolution FT detectors, including the Orbitrap (achievable resolution >200,000) and Ion Cyclotron Resonance detectors with resolutions >500,000.

Ion mobility spectrometry, when coupled to MS, is a hybrid approach that first separates ions by their mobility in a carrier gas on a millisecond timescale, followed by detection with a MS (Jackson et al. 2014b; Stauber et al. 2010). The hyphenated approach offers the ability to separate ions with similar *m*/*z* but different shapes, providing the added benefits of an orthogonal separation phase within the single acquisition instrument. Benefits include better signal-to-noise (S/N) ratio and the potential to separate isomers according to their shape and charge. There are a number of reported MSI applications imaging lipids in a variety of mammalian tissues (Jackson et al. 2014b; Stauber et al. 2010). The approach is yet to be applied to plants.

## Multimodal imaging

**Fig. 4:**
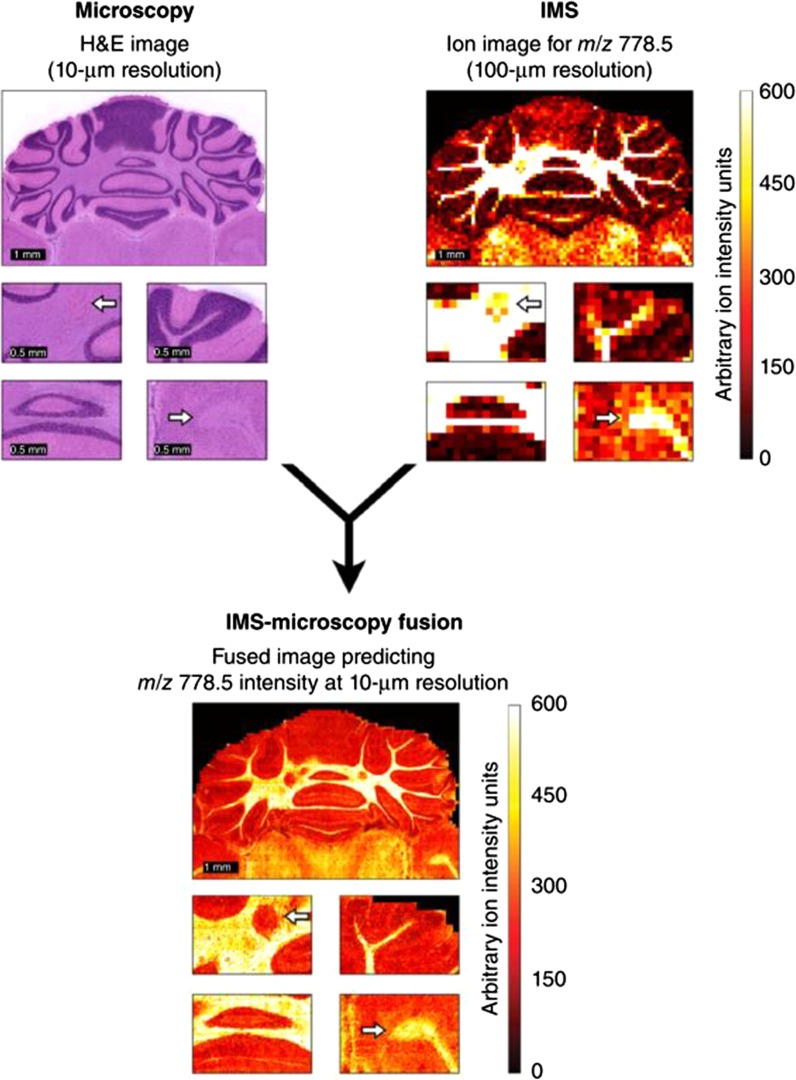
Demonstrates the image fusion approach combing two different image modalities at differing lateral resolutions. By using information contained in the higher lateral resolution image the distribution of a lipid can be predicted. Example of IMS-microscopy fusion. An ion image measured in mouse brain, describing the distribution of m/z 778.5 [identified as lipid (PE(P-40:4)] at 100 µm spatial resolution (*top right*), is integrated with an H&E microscopy image measured from the same tissue sample at 10 µm resolution (*top left*), by combing the information from both image types, the image fusion process can predict the ion distribution of m/z 778.5 at 10 µm resolution (*bottom*). Reprinted by permission from 1629 Macmillan Publishers Ltd: Nature Methods, (Van de Plas et al., 2015) 12(4):366-72, Copyright © 2015

Within the molecular imaging field, the use of different types of imaging modalities to examine a single biological question is common. However, the different datasets are typically treated as separate entities. An emerging theme is multi-modal imaging, which involves combing two or more imaging modalities; a common approach is to generate a histochemically stained section of tissue, either a serial section or in some cases the same piece of tissue on which an MSI measurement has been conducted. Co-registration of high-resolution optical images from histochemical staining with the acquired MSI data provides more in-depth information (tissue/cell type distribution), aiding sample interpretation. Another combination is that of MALDI and SIMS, which has been used extensively in plant and animal MSI imaging (Chughtai et al. 2012; Hanrieder et al. 2014; Ogrinc Potočnik et al. 2014; Seaman et al. 2014), where the former has been used to generate lower-resolution images across a wide area, and SIMS for very-high-resolution imaging of a smaller sub-section of the tissue. The modality need not be another MSI technique or optical imaging approach; other modalities including MRI, FT-IR and XRF could be employed to examine the underlying biology. Previously, MSI was combined with high-resolution magnetic resonance spectroscopic imaging (MRSI) to examine choline metabolites and cations in tumor cells (Amstalden van Hove et al. 2010). Recently, a hybrid predictive technique called Image Fusion has been reported (Fig. 4). The approach uses the combination of information containing high spatial resolution but low chemical specificity, such as images generated from optical microscopy at high magnification, coupled to lower-spatial resolution but high chemical specificity information, such as MSI data, to computationally predict the distribution of chemicals in the tissue sections. The approach has a number of advantages and in principal can be applied to and fuse a variety of different imaging modalities (Van de Plas et al. 2015).

## Advantages and limitations of MSI

MSI has a number of advantages over other imaging modalities, which are directly derived from the capabilities of modern mass spectrometers. MSI provides high molecular selectivity, high sensitivity and rapid multiplexed acquisition of many ionizable compounds in a single measurement. MSI can provide very high lateral resolutions for imaging, giving the ability to distinguish the molecular nature of fine morphological features of tissues. In practice, lateral resolutions of 50 nm for nano-SIMS, 10–50 µm for MALDI instruments with front-side illumination geometry, 1 µm for transmission geometry MALDI instruments and between 15 and 250 µm for nano-DESI and DESI type ion sources can be achieved. Many of the approaches allow lateral resolutions of around the size of a mammalian cell (~10 to 30 µm) or subcellular resolution for large plant cells (~10 to 100 µm). Advanced electron multipliers and ion detectors impart the capability to measure very low numbers of ions, providing extremely high sensitivity for individual ions. When MSI is coupled to accurate mass analyzers, MSI has a unique ability to distinguish many compounds at once in a profiling-type experiment. This would not be possible with a low-resolution instrument, however, the same instruments operated in MS/MS mode can provide very high selectivity and sensitivity using SRM/MRM or ion trapping techniques. The application of ion mobility separation to MSI experiments holds promise for the analysis of isomers and closely related metabolites, particularly large lipid molecules, peptides and proteins. However, the benefits of ion mobility for small molecule analysis are still being assessed. The capacity of modern MSI instruments and software allows rapid collection of data that can allow high-throughput analysis and screening approaches.

There are a number of limitations of MSI, including the ion suppression and space charging effects for ion trap-type instruments (LIT, LIT-TOF, FT-ICR, Orbitrap). When operating MSI instruments at very high spatial resolutions, there is a significant trade-off with sensitivity, as the total number of ions decreases with the sampling area, and thus the overall sensitivity for individual ions will be compromised. MSI experiments are less sensitive than analyses that utilize an orthogonal separation prior to measurement and detection; this is directly due to the extremely complex biological matrix of the tissues where vast concentration ranges of chemical entities are present with differing chemistries and molecular sizes (e.g. proteins, lipids, organic acids, amino acids, carbohydrates, inorganic ions etc.). The generation of competing ions from chemicals with vastly different desorption and ionization efficiencies from the same sampling area leads to an ion suppression effect, where ions that may be preferred or those that are present in higher abundance dominate over low-abundance or poorly ionizable molecules. For MALDI experiments, the presence of high-abundance low-weight ions generated directly from the matrix leads to significant interfering signals. MSI is generally an ex vivo technique that relies upon harvesting appropriate tissue samples from biological organisms for analysis. This is in contrast to in vivo imaging modalities (MRI, PET, X-ray, XRF) that do not require tissue harvesting. During sample harvesting, preparation and analysis, there are many potential pitfalls that must be avoided to obtain useful images; any sample preparation steps or techniques have the potential to contaminate the tissue section with exogenous material that may affect reproducibility, ionization and image quality, and that may complicate the data analysis. Finally, the most significant bottleneck of MSI experiments remains the lack of robust and efficient data analysis pipelines, particularly for analysis of ultra-high resolution FT data. The recent adoption of common data output standards, the ongoing development of software, and a move towards common standards will likely address many aspects of these problems in the very near future (see below).

## Sample preparation

Prior to analysis, tissues must be collected and stored. The steps taken during both tissue collection and storage are critical for successful MSI analysis and often vary depending upon the analyte of interest. Most experiments will have a distinct timing miss-match between sample collection and analysis, requiring the storage of samples for a period of time. Typically, to protect delicate tissues, structures and small metabolites, a gentle freezing approach is recommended, including freezing in the atmosphere over liquid nitrogen or in cold carbon dioxide atmosphere over dry-ice, alternatively samples may be dipped into isopentane:liquid nitrogen or isopentane:dry-ice slurries. Tissue stabilization methods for proteins include heat stabilization and microwaving (Goodwin et al. 2008, 2010, 2012). For previously fixed tissue samples, there are a number of sample preparation protocols that have been developed for Formalin-Fixed Paraffin-Embedded (FFPE) mammalian tissue specifically for MSI analysis (Powers et al. 2014). Although these methods have not yet been tested for plant samples, the developed protocols are likely to be directly applicable to formalin-fixed plant tissues, noting that the fixation approach is only suitable for examination of metal distribution, proteins, peptides and other polymeric biomolecules in tissues, due to the fixation process typically leading to the extraction and degradation of small molecules. Further tissue preparation steps for this process are lengthy due to the need to conduct antigen retrieval steps. For most MSI analyses, tissue samples are typically flash-frozen to quench metabolism and retain the spatial distribution of analytes. Care must be taken to retain the tissue morphology during the freezing process and to preserve an accurate representation of the native tissue; soft tissues may deform and take the shape of the container (tube or tray) within which they are frozen. Once frozen, tissues and analytes are generally stable for months to years when stored at −80 °C. Embedding tissues within an external matrix is a common approach and is often required to ensure that suitable sections are generated from fragile frozen plant tissues which have a distinct tendency to fracture and crumble. A number of different embedding media have been successfully demonstrated, including agarose (Marques et al. 2014), gelatin (Gemperline and Li 2014a; Horn et al. 2013b, 2014; Korte and Lee 2014; Korte et al. 2015; Ye et al. 2013) and aqueous carboxymethyl-cellulose solutions (1–5 %) (Bencivenni et al. 2014; Yoshimura et al. 2012b). In general, the easier the frozen matrix is to section or the closer the properties of the matrix are to the tissue being sectioned, the easier it will be to generate suitable sections of tissue for analysis. Thicker plant tissue sections (of 20–50 µm) than for mammalian tissues (5–25 µm) are recommended to limit fracturing and crumbling. Standard histological workflows utilize Optimal Cutting Temperature (OCT) compound [a solution containing ~4 % poly-ethylene glycols (PEG)] as an embedding medium, but this is strongly discouraged for MSI research due to absorption into the tissue and smearing of OCT across the tissue surface during cryo-sectioning, which has been shown to directly lead to ion suppression effects and loss of analyte signals (Schwartz et al. 2003).

Depending on the analysis method and instrument used, tissues must be prepared differently for imaging purposes, and a number of factors must be considered. External surfaces can be readily analyzed by mounting tissues directly to sample stages using double-sided tape, but for the measurement of internal distributions of metabolites tissues must first be sectioned at an appropriate thickness to expose the underlying tissue. In particular, the type of analytes and their stability and turnover must be considered. To retain metabolite distributions and concentrations, tissues are flash frozen during harvesting and sectioned or prepared at a later time point. Both the sample height and morphology may have a large effect upon the number of ions generated (due to laser focusing) and, for linear TOF instruments (LDI and MALDI), mass accuracy and resolution (due to changes in flight path length). Instruments where the detector is decoupled from the source, such as LIT, FT-ICR and Orbitrap instruments, are not reliant upon the sample thickness and are only limited by the physical configuration of the sample stage.

While cryo-sectioning is the most commonly used method for sample preparation to access internal metabolites, there are other alternatives for tissue sectioning. For ambient ionization techniques such as LTP-MS, AP-IR-MALDI-MS, DESI-MS and LAESI-MS, the tissue thickness is reliant upon the sample stage configuration used for MS measurement. Manual tissue sectioning is practiced using a knife blade, vibratome or microtome with thickness of 200 µm upward to 4 mm without freezing (Li et al. 2007; Maldonado-Torres et al. 2014; Tata et al. 2015; Vaikkinen et al. 2013). In addition, a hollow punch has been used to produce grapevine leaf discs (Becker et al. 2014). Tissue samples are normally frozen to preserve their native metabolic state and prevent the loss of metabolites with short half-lives. While DESI mainly analyses freshly frozen tissues, fresh non-frozen tissues are used in many other methods. Very recently, a fracturing approach has been demonstrated for accessing the internal cell layers of rice leaf (Klein et al. 2015). For surface metabolite analysis, samples are used directly for MS analysis without pre-treatment. However, analysis of high-molecular-weight plant cell surface/wall components, such as cutin, suberin, acetylated arabinoxylan and beta-glucans, requires in situ chemical (e.g. alkaline hydrolysis) or enzymatic (lichenase and xylanase) digestions, in order to depolymerise the polymers prior to MS analysis (Velickovic et al. 2014; Veličković et al. 2014).

For ionization techniques and stages that are under high vacuum, e.g. in SIMS and MALDI-TOF instruments, the instrument configuration typically requires a number of extra steps during sample preparation. For these techniques, tissue thickness is typically 8–50 µm, but can be up to 1.5 mm. Once mounted to the sample carrier, the tissues are typically dehydrated under vacuum prior to either matrix deposition or direct analysis. Prior dehydration avoids any shrinkage of tissues leading to changes in sample morphology within the instrument. In MALDI-MS using TOF detection, where a voltage is applied to the sample stage, samples are usually mounted either on glass slides coated with conductive indium tin oxide (ITO), or on re-useable metal sample stages (steel or gold-coated steel). Samples are either directly freeze–thaw mounted to the surface or adhered using conductive double-sided tape (Burrell et al. 2007). Freeze–thaw mounting is generally performed by transferring the cut tissue section to the top of the sample holder (slide, plate), then gently warming the holder from the underside using body heat. The tissue section quickly thaws and adheres to the surface of the holder. Once mounted, the sections are warmed and transferred to a vacuum desiccation chamber and dried under reduced pressure for at least 15 min before any further steps are conducted. Tissue sections may degrade rapidly and must either be stored under vacuum or, for longer periods, at −80 °C (Patterson et al. 2014). For MALDI-MSI, application of the matrix has been shown to stabilize analytes within the tissue to oxidation and degradation processes.

Other ionization sources that do not require a voltage to be applied to the sample carrier, such as AP-SMALDI-MS, LAESI-MS, LAAPPI-MS, LTP-MS and SIMS, generally use normal glass slides (disposable), metal or silicon surfaces. To prevent inaccurate analysis of uneven samples, imprinting techniques are common, especially in DESI-MS, using either paper, TLC or PTFE substrates (Ifa et al. 2011; Lane et al. 2009; Li et al. 2011; Muller et al. 2011; Thunig et al. 2011), although glass slides (Andras et al. 2012) and tapes (Tata et al. 2015) have also been reported. Similarly, detection of trace elements via LA-ICP-MS requires samples to be fixed onto acetate double–sided adhesive tape, before placing them into an ablation chamber (da Silva and Arruda 2013).

### Tissue washing

A commonly accepted principle of MSI analysis is to conduct the minimal amount of sample preparation steps, to avoid metabolite degradation and retain the distribution of analytes. However, a number of tissue washing steps can be conducted to either increase the sensitivity for certain analytes or to remove background salts to decrease salt adducts (Angel et al. 2012; Seeley et al. 2008; van Hove et al. 2011). Mounted sections can be carefully dipped into washing solutions and then dried, before further processing such as enzymatic digestion or application of matrix. These steps have been successfully employed to increase the ionization of selected metabolites (including lipids, proteins and peptides) in mammalian systems, but have not yet been demonstrated in plant systems.

### MALDI matrix application and in situ protein digestion strategies

MALDI and Matrix-Enhanced SIMS techniques rely upon an exogenous matrix, consisting typically of either small organic molecules or inorganic UV absorbent nano-particles, which must be applied by one of a number of different techniques. Further, the achievable lateral resolution is dependent upon the size of the matrix crystals, which is in turn dependent upon the application technique employed. There are a number of approaches used to apply a MALDI matrix that can be separated into two different strategies, involving either dry deposition or wet deposition and extraction. The first, dry deposition strategy, deposits the matrix without any solvents to the top surface of a tissue section by one of two common techniques, employing hand-shaking of dry fine crystals of matrix onto the sample through a sieve or the use of a sublimation apparatus. A sublimation approach for deposition of matrix provides very uniform coatings with very small crystal sizes (typically in the range of 1–5 µm), allowing imaging with high spatial resolution. It is becoming one of the preferred approaches for small-molecule and lipid imaging (Hankin et al. 2007).

Wet deposition strategies have also had significant attention, and there are many different techniques available for specific analyte classes. Wet deposition is one of the most common techniques for matrix deposition for MALDI-MSI analysis and is essential to conduct in situ protein digests. To conduct an in situ protein digestion, a protease, generally trypsin or α-chymotrypsin, is deposited in a buffered solution. Once uniform application of enzyme has been achieved, the sample is incubated in a humid atmosphere for a period of time, to allow localized digestion before drying and matrix application for MALDI-MSI. As yet, no in situ protein digestion strategies have been reported in plant-based MSI. Matrix is first dissolved in a suitable solvent, then small droplets are applied to the surface of the tissue to be imaged, micro-extraction of endogenous molecules takes place at the solvent-tissue interface and, as the solvent dries, analytes co-crystallize with the dissolved matrix. The achievable lateral resolution of a wet deposition technique is predominantly dependent upon the droplet size maintained during matrix deposition. There are several different techniques reported in the literature, including home-made solutions and a range of commercially available instruments, ranging from manual airbrushing (where success is highly dependent upon the operator) to more controlled robotic spraying (HTX Imaging TM-Sprayer, HTX Technologies LLC, Carrboro, NC, USA; SunChrom SunCollect and SunCollect II plus+, SunChrom GmbH, Friedrichsdorf, Germany), automatic droplet deposition through piezoelectric vibration (ImagePrep, Bruker, Bremen, Germany), inkjet printing (ChIP 1000, Shimadzu Corp., Japan) with standard inkjet printers (Baluya et al. 2007), robotic spotting (Labcyte Portrait 630 Spotter—no longer available) and automatic protein digestion robots (SunChrom SunDigest, SunCollect II plus+, SunChrom). Once deposition conditions have been optimized for specific solvents, matrix and concentration, number of passes or spray cycles, temperatures and drying, it is possible to achieve very small crystal sizes of 5–20+ µm (in the longest dimension), allowing high-resolution imaging. A combination approach of initial dry deposition using sublimation followed by in situ ‘rehydration/recrystallization’ by vapor exchange provides excellent results for protein and peptide imaging (Norris and Caprioli 2013).

### Matrices for MALDI analysis

There are a large number of matrices that are either in common use or have been recently reported in the literature for MALDI, including the main stalwarts 2,5-dihydroxybenzoic acid (DHB) (Becker et al. 2014), 2,5-dihydroxyacetophenone (DHAP) (Meriaux et al. 2010), sinapinic acid (SA) (Anderson et al. 2015; Fraser et al. 2007), and α-cyano-4-hydroxycinnamic acid (CHCA) (Debois et al. 2014; Franceschi et al. 2012; Gemperline and Li 2014b), which are typically used for positive mode MALDI analysis. 9-aminoacridine (9-AA) (Korte and Lee 2014; Shroff et al. 2015), 1,8-bis-dimethylaminonaphthalene (DMAN) (Horn et al. 2012; Ye et al. 2013) and 1,5-diaminonaphthalene (DAN) (Becker et al. 2014; Korte and Lee 2014; Korte et al. 2015) were reported for negative mode analyses. 2-aminoethyl-*N*-2-aminonaphthalene has also been reported as a suitable matrix (Cha et al. 2008). Recent use of the plant metabolites quercetin and morin (Wang et al. 2013), which are structural isomers, as matrices for both positive- and negative-mode analysis has demonstrated vastly increased detection of phospholipids in mammalian tissues when using high-resolution FT-ICR-MS. The use of plant-based flavonols as a MALDI matrix will require careful assessment for each system being imaged, including the method for deposition (wet vs. dry) and the analytes being targeted, to avoid any complications. Indeed, it is the natural abundance of endogenous flavonols in high concentrations in plants, together with other UV-absorbing metabolites, that allows UV-LDI approaches to be employed in these systems. In these cases, the plants’ own metabolites are acting as an endogenous MALDI matrix (Holscher et al. 2009). Some caution must be employed if the distribution of UV-absorbing molecules in the plant tissues is not uniform, since then the ion yield from each raster may be different, likely yielding misleading results.

More recently, DAN has been adopted for plant-based imaging, which requires very low laser energy and very small crystal size (Korte and Lee 2014). DAN has been used for MSI imaging in both positive and negative modes at very high spatial resolution. (However, caution is required when using DAN as it is suspected to be a carcinogen.) Further, DAN is also chemically reactive with the ability to form gas phase radicals, to induce in-source decay and to conduct gas phase reductions of disulphide bonds (Molin et al. 2011; Yang et al. 2009). The use of an ambient-pressure MALDI source allows the use of volatile matrices, including liquid ion matrices and also water in the form of ice for IR-MALDI within frozen tissues (Robichaud et al. 2014). Nanoparticles and colloids have been reported as suitable matrices for MALDI-MSI, including the use of silver and gold nano-particles for the imaging of waxes and phospholipids (Dufresne et al. 2013; Jackson et al. 2007, 2014a; Muller et al. 2015; Wu et al. 2009). Furthermore, functional iron nano-particles (fNPs) have been demonstrated in mammalian tissues (Taira et al. 2008). More recently, silica particles have been reported as a suitable matrix for the analysis of lignin oligomers in *Eucalyptus* species, and colloidal graphite in a Graphite-Assisted LDI approach (GALDI) for imaging plant metabolites (Cha and Yeung 2007). In the case of small-molecule matrices, these can be readily removed post-MSI acquisition, washed with a suitable solvent such as ethanol or aqueous solutions, and then subjected to histochemical staining (Norris and Caprioli 2013).

## Identification and quantification strategies

Due to the nature of an MSI experiment, it is not always possible to determine exact chemical structures from single-stage accurate-mass information acquired by MSI. Typically, an orthogonal analysis is required to increase both the depth of coverage and the sensitivity and allow identification of as many chemical species as possible. The presence of either isomers (structural or stereo) or near-isobaric chemicals confounds the interpretation of the spectra. Near-isobaric chemicals can be distinguished by the use of higher mass-resolving power, which at the extremes can be used to provide unambiguous identification of molecular formula by measurement of the isotopic fine structure at RP > 300,000 FWHM (Miura et al. 2010). The high mass RP required is only achieved using FT instruments, including FT-ICR and the latest Orbitrap MS instruments. However, the identification of molecular formula still does not provide absolute identification of isomeric compounds; instead, MS/MS (de Hoffmann 1996) can be used to identify individual chemicals by fragmentation analysis, where precursor ions are fragmented using techniques including Collision-Induced Dissociation (CID) (Sleno and Volmer 2004), Higher-Energy Collisional Dissociation (HCD) (McAlister et al. 2011; Olsen et al. 2007), Electron Transfer Dissociation (ETD) (Syka et al. 2004), Electron Capture Dissociation (ECD) (Zubarev et al. 2000), or Sustained Off-Resonance Collision-Induced Dissociation (SORI-CID) (Gauthier et al. 1991). Individual compounds fragment in a unique manner, generating combinations of product ions whose relative abundances can be compared to those of an authentic standard to identify the precursor ion. Identification of individual metabolites may be difficult due to low fragment ion abundance or interfering ions. To increase sensitivity, SRM- and MRM-type experiments using instruments capable of MS/MS can be employed, but the tradeoff is an inability to monitor wide mass ranges (Barry et al. 2014). There are a number of on-line databases that include high-resolution mass spectra and also in some cases MS/MS fragmentation patterns of endogenous and exogenous molecules, making them useful for identifying metabolites, including: METLIN (https://meltlin.scripps.edu), LIPIDMAPS (www.lipidmaps.org), MassBank (www.massbank.jp), Human Metabolome Database (HMDB, www.hmdb.ca) and mzCloud (www.mzcloud.org). As most spectral databases have been generated for human systems, the total number of spectra for plant metabolites is low. Further compound databases can be searched for accurate mass and formula matches, including PubChem, ChEBI, and the In Vivo/In Silico Metabolites Database (IIMDB) (http://metabolomics.pharm.uconn.edu/iimdb/).

Quantitation and validation of analytes pixel-by-pixel in MSI datasets is challenging and is severely affected by the local tissue environment or ‘matrix effect’, evident as ion suppression effects from the complex series of chemicals present (see Ellis et al. 2014a for a detailed review). Typically, an orthogonal or complementary approach is conducted to provide absolute quantitation (Berisha et al. 2014; Tomlinson et al. 2014). There have been several approaches to quantitation, including the generation of external standard curves by doping of tissue homogenates or direct application of a dilution series onto the tissue section. This approach is most suitable for exogenous materials, including drugs and contaminants; endogenous metabolite quantitation is a more challenging problem and requires the use of labelled internal standards. The commercial software Quantinetix™ (ImaBiotech, Lille, France) is available to conduct quantitative MSI experiments and has seen extensive use for studying drug distribution, pharmacokinetics and toxicology. For LA-ICP-MS systems, a quantitative approach has been demonstrated for the imaging of metal ions in tissues using a single-point calibration (Sussulini et al. 2013).

## Analytical software and data analysis techniques

Due to the sheer volume and complexity of the data generated in MSI experiments, there is a requirement for advanced software and computational data analysis techniques to extract meaningful results from the data. Originally, data analysis of MSI datasets was largely limited to manual identification and mapping of individual ions; analysis software had not yet incorporated advanced clustering and comparative visualization tools allowing spatial segmentation, identification and comparison of multiple ions. The need for advanced software to analyze MSI datasets drove the generation of a number of software packages, and over the past 3 years there has been significant development and improvement in the range of software. This includes BioMap (Novartis, Basel, Switzerland), Datacube Explorer (FOM-AMOLF, Amsterdam, Netherlands) (Klinkert et al. 2014), FlexImaging and ClinProTools (Bruker Daltonik, Bremen, Germany), HDI (high-definition MALDI MS imaging) coupled to MassLynx and MarkerLynx (Waters, Manchester UK), ImageQuest (Thermo Scientific, Waltham, MA, USA), MALDIVision (PREMIER Biosoft), Metabolite Imager (University of Texas) (Horn and Chapman 2013), MIRION (Justus Liebig University) (Paschke et al. 2013), MSiReader (North Carolina State University) (Robichaud et al. 2013), OpenMSI (Lawrence Berkeley National Lab, CA, USA, http://openmsi.nersc.gov) (Rübel et al. 2013), SCiLS Lab (SCiLS Bremen, Germany) and TissueView (AB Sciex, based on BioMap). The website maldi-msi.org, operated by a consortium of European MSI users, provides a number of free software tools for download. Many researchers still rely on “in-house” data analysis methods, and the use of MATLAB tools is common. Very recent adoption of the common imzML data format standard (www.imzml.org) (Schramm et al. 2012) by instrument vendors and incorporation into a variety of tools or directly into the vendor software (such as FlexImaging) has allowed export of instrument-specific data into a common format, which has aided the development of vendor-independent tools for data analysis and application of advanced statistical techniques to identify underlying metabolite distributions and co-localizations. Many of the current packages for MS image analysis have been developed incorporating only visualization and simple clustering techniques such as Hierarchical Cluster Analysis (HCA) and Principal Component Analysis (PCA).

Due to the inherent heterogeneity of MSI data, pre-processing and spectral “de-noising” is recommended to obtain better results (Alexandrov 2012; Alexandrov et al. 2013; Norris et al. 2007). Pre-processing includes steps for baseline subtraction and smoothing, peak alignment and mass recalibration across the entire dataset, normalization of signal intensity, peak-picking and data reduction steps. A number of publications have provided detailed analysis pathways and suitable tools to examine MSI data (Alexandrov 2012; Rübel et al. 2013). Once pre-processing steps are complete, there are three types of unsupervised approaches to identify hidden patterns and spatial distributions of metabolites: Component Analysis, Spatial Segmentation and Self-Organizing Maps. The first, component analysis, has been dominated by the use of Principal Component Analysis (PCA), although other methods have been used to uncover the variation in MALDI-MSI data, including non-negative matrix factorization, maximum auto-correlation factorization and latent semantic analysis [see review by Alexandrov (2012) for further details]. PCA represents the spatial patterns of molecules in terms of the set of score images, but PCA has a number of limitations including negative values (which are not present in the data) and difficulty in determining co-localized ion images for identified patterns of distribution. Spatial segmentation is a robust approach to examine MSI data where a segmentation map displays different regions in the tissues with distinct molecular composition (Alexandrov 2012). A common approach is to use Hierarchical Cluster Analysis (HCA), which is directly incorporated into FlexImaging. More recently, advanced spatial segmentation clustering techniques have been developed that cluster *m*/*z* values with distinct regions of the tissue (Alexandrov et al. 2013; Krasny et al. 2015) and are incorporated directly into the commercial software SCiLS Lab. The third area is an emerging data analysis technique that makes use of unsupervised Self-Organizing Maps (SOM) (Fonville et al. 2013; Franceschi and Wehrens 2014) and Growing Self-Organizing Maps (Wijetunge et al. 2014) that reduce the dimensionality of the data and allow identification of hidden patterns within the data.

Three-Dimensional Mass Spectrometry Imaging (3D-MSI) has been reported (Andersson et al. 2008) and reviewed previously (Seeley and Caprioli 2012). 3D-MSI is conducted using one of two approaches: (1) depth profiling on the same tissues by conducting sequential rastering events (Seeley and Caprioli 2012), which is common for SIMS (Fletcher et al. 2011a, b) but has also been reported for LAESI which was used to depth profile plant leaf tissue (Nemes et al. 2009); or (2) by combining multiple two-dimensional MSI measurements conducted on serial tissue sections from a single sample. Individual datasets are computationally re-assembled to generate 3D volume reconstructions of individual ion distributions; for this purpose, researchers have used software such as Amira (www.fei.com), Image J (http://imagej.nih.gov/ij), MATLAB (www.mathworks.com) and more recently SCiLS Lab (scils.de) to generate 3D images.

## Common reporting standards and repository

Recent guidelines for the reporting of MSI datasets have been published (McDonnell et al. 2015). The article outlines the detailed metadata and contextualizing of information that is required to fully describe a MSI dataset, and it provides eight specific reportable areas: (1) Tissue Samples, including the type and how the tissue was sampled; (2) Tissue Preparation, including methods such as washing and matrix application steps; (3) Optical Image, detailing information about the corresponding optical images used for MSI analysis; (4) Data Acquisition, detailing the instrument and parameters used to acquire the data; (5) Mass Spectra Pre-processing, detailing the parameters used to baseline subtract, to smooth and align spectra, for intensity normalization methods, for peak picking and data reduction methods; (6) MSI Visualization, including methods for peak picking and image generation parameters; (7) Compound Identification, including all procedures used to identify individual metabolites; and (8) Data Analysis, detailing procedures, methods and software used. The reader is also reminded that there are a number of other reporting standards for identification in metabolomics experiments, including definitions for tentative, putative and confirmed identification (Creek et al. 2014). For MSI experiments, the ability to confirm identifications is all the more difficult, due to the inability to separate isobaric compounds. A common public repository has also recently been announced, where MSI datasets can be deposited for storage and later retrieval, although the repository may be more suited to biomedical and clinical datasets (Rompp et al. 2015).

## Applications

The types of publications focused on plant MSI are evolving; at the beginning, subject matter tended to cover the application of a new technology to plants and the development of new techniques and methods to examine plant metabolites (see Table 3 for a complete list of plant-based MSI publications). Although there is still much technical development required, the approach has matured to a point where more advanced questions in plant biology are now being addressed. There are a number of common themes emerging that range from fundamental biology, plant structural and surface metabolites and plant responses to abiotic and biotic stresses, plant pathology and symbiont systems. The targets of analysis have typically been highly abundant plant metabolites that include natural products, structural components and their precursors, defense and energy storage molecules.

**Table 3:** Table of MSI plant based publications by instrumental approach and year until April 2015, including: species, sample type (tissue), MSI technique and image resolution, imaged analytes, sample preparation protocols, reference in this paper, orthogonal identification strategy

Year	Species	Sample type	MSI technique and image resolution	Imaged analytes	Sample preparation	References	Identification strategy
*Desorption Electrospray Ionisation*							
2009	Red macroalga (*C. serratus*)	Blade	DESI, 200 µm	Bromophycolides	Mounting on PTFE substrate	Lane et al. (2009)	1H NMR, LC–MS, ESI–MS
2011	St. John’s wort (*H. perforatum*); thorn apple (*Datura stramonium*); opium poppy (*Papaver somniferum*)	Leaf, petal, capsule	DESI, 100–125 µm	Phloroglucinols, flavonoids, naphthodianthrones, saccharides, alkaloids	Imprinting onto porous PTFE	Thunig et al. (2011)	MS/MS
2011	Barley (*Hordeum vulgare*)	Leaf	DESI, 100–200 µm	Hydroxynitrile glucosides	Stripping of epidermis or imprinting onto porous PTFE	Li et al. (2011)	MS/MS
2011	Katsura tree (*C. japonicum*) and American sweetgum (*Liquidambar styraciflua*)	Leaf	DESI, 130–310 µm	Chlorophyll catabolites	Imprinting onto porous PTFE	Muller et al. (2011)	MS/MS
2011	*Myristica malabarica*	Seed	DESI, 250 µm	Alkaloid	Cross-sectioning and imprinting onto printer paper	Ifa et al. (2011)	–
2012	Red alga (*Phacelocarpus neurymeniodides*)	Blade	DESI, 180 µm	Antibacterial metabolite neurymenolide A	Mounting to glass slides with glue, followed by direct DESI imaging	Andras et al. (2012)	1H and 13C NMR; HPLC–MS
2013	Potato (*Solanum tuberosum*), Gingko (*Gingko biloba* L.), Strawberry (*Fragaria* × *ananassa* Duch.)	Leaf, fruit	DESI, 150–200 μm	Glykoalkaloids, flavooids, sugars and anthocyanidin	Manual cross section and imprinting on TLC plates or glass slides	Cabral et al. (2013)	
2015	Potato sprout (*Solanum tubersum*)	Tuber	DESI, 150–200 μm	Glycoalkaloids	Samples were sectioned using a sterile knife, imprinted on tapes and mounted using double sided tape	Tata et al. (2015)	MS/MS (CID)
*Laser Ablation*							
2007	French marigold (*Tagetes patula*)	Leaf, stem, and root	LAESI	Primary and secondary metabolites	Sample mounted on microscope slides	Nemes and Vertes (2007)	–
2008	Zebra plant (*Aphelandra squarrose*)	Leaf	LAESI, 350 µm	Primary and secondary metabolites	Mounting of sample on glass slides	Nemes et al. (2008)	–
2009	Peace lily (*Spathiphyllum lynise*) and zebra plant (*Aphelandra squarrose*)	Leaf	LAESI (3D), 300 µm lateral, 30–40 µm depth	Secondary metabolites	Mounting on glass slides	Nemes et al. (2009)	–
2011	Onion (*Allium. cepa*) and sour orange (*Citrus aurantium*)	Bulb, leaf	LAESI, ∼30 μm	Metabolites	Layer of onion bulb scales was excised by a surgical scalpel into a strip. Intact layer of the inner epidermal tissue was peeled away and mounted onto a glass slide. Sour orange leaves were excised and secured to glass slides with tape	Shrestha et al. (2011)	MS/MS
2012	Sour orange (*Citrus aurantium*)	Leaf	LA-APPI, ∼300 μm	Polar and nonpolar compounds	Samples attached onto a microscope glass slide with adhesive tape	Vaikkinen et al. (2012)	–
2013	Aavocado (*Persea americana*), Pansy (*Viola*)	Mesocarp, petal	LAESI, HA-LAESI, LA-APPI	Nonpolar and polar compounds	Avocado sample was cut with a blade (10 × 20 × 0.5 mm) and placed on a microscope glass slide using a manual microtome. Pansy flower petals were attached to a glass microscope slide using adhesive tape without any pretreatment	Vaikkinen et al. (2013)	–
*Laser Ablation Inductively Coupled Plasma*							
2013	Sunflower (*Helianthus annuus*)	Leaf	LA-ICP	Selenium (Se), sulphur (S)	Samples fixed onto acetate double-sided adhesive tape and placed into ablation chamber	da Silva and Arruda (2013)	ICP-MS
*Laser Desorption Ionisation*							
2007	Apple (*Malus domestica*), Strawberry (*Fragaria* × *ananassa*)	Fruit	GALDI, 100 µm	Organic acids, flavonoids and oligosaccharides	Cryo-sectioning (15 µm), mounting on stainless steel plate	Zhang et al. (2007)	MS/MS
2008	Thale cress (*Arabidopsis thaliana*)	Flowers, petals, leaves, stem	GALDI, 100 µm	Flavonoids, cuticular waxes	Cryo-sectioning, double sided tape for leaves and flowers	Cha et al. (2008)	MS/MS
2009	Ginger (*Zingiber officinale* Roscoe)	Rhizome	AP-LDI, 10–20 µm	Gingerol, terpenoids, saccharides	Manual section using razor blade, mounted onto ITO slides using double sided conductive tape	Harada et al. (2009)	MS/MS
2009	Thale cress (*Arabidopsis thaliana*); St. John’s wort (*H. reflexum* and *H. perforatum*)	Stamen, petal, leaves, placenta, pollen	LDI, 10 µm	Secondary metabolites	Mounting with carbon conductive adhesive tape. Laser micro-dissection (stigma), cryo-sectioning (placenta, 60 mm)	Holscher et al. (2009)	–
2009	Thale cress (*Arabidopsis thaliana*)	Flower, leaf	LDI, 50–100 µm	Epicuticular wax metabolites	Fixing to stainless steel plate with conductive double sided tape, drying. Coated with colloidal silver solution	Cha et al. (2009)	GC–MS
2010	Thale cress (*Arabidopsis thaliana*)	Flower	LDI, 12 µm	Epicuticular wax and alkyl ester metabolites	Samples attached onto a stainless steel plate using conductive double-sided tape. Coating with colloidal silver and colloidal graphite	Jun et al. (2010)	GC–MS
2010	Grape vine (*Vitus vinifera*)	Leaf	LDI, 25 µm	Stilbenoids	Mounted to MALDI plate with aluminized tape	Hamm et al. (2010)	–
2010	Switchgrass (*Miscanthus giganteus*)	Stem	LDI/MALDI, 100 µm; SIMS, 2 µm, 22 keV Au1+ beam	Saccharides	Cryo-sectioning (50 µm) LDI: Thaw mounting on glass slides. No matrix, DHB or CHCA matrix. Coating with gold. SIMS: Thaw mounting on Si wafer, drying, coating with gold	Li et al. (2010a, b)	–
2012	Thale cress (*Arabidopsis thaliana*)	Flower	LDI/MALDI	Flavonoid	Petals and whole flowers were mounted on stainless steel sample plates with conductive double-sided tape. No matrix or colloidal matrix	Korte et al. (2012)	LC/MS
2014	Wild Daisy plants (*Lychnophora salicifolia, L. ericoides* and *L. pinaster*)	Leaf	LDI, spatial resolution not provided	Flavonoids	Samples were sectioned using microtome (50 μm), adhered to ITO slides using double sided tape	Silva et al. (2014)	UPLC-MS/MS, LDI-MS, LDI-MS/MS
2014	Banana (*Musa* spp.)	Root	LDI, 10 μm	Phenylphenal-enones	Samples cryo-sectioned and fixed on carbon-conductive adhesive tape and fixed on ITO slides	Hölscher et al. (2014)	1H NMR, Raman microspectroscopy, HPLC
*Low Temperature Plasma*							
2014	Chili pepper (*Capsicum spp.*)	Fruit	LTP, 1 mm	Capsaicin	Longitudinal cross-section of sample (80 × 35 × 4 mm) laid directly onto a glass slide fixed on a sample carrier	Maldonado-Torres et al. (2014)	–
*Matrix Assisted Laser Desorption Ionisation*							
2005	Soya (*Glycine max*)	Leaf, stem	MALDI	Mesotrione and azoxystrobin (pesticides)	Freeze-dried mounted with conductive tape or blotting onto acetone wetted cellulose membrane. CHCA matrix	Mullen et al. (2005)	–
2007	Strawberry (*Fragaria* × *ananassa*)	Fruit skin	AP IR-MALDI, 200 µm	Saccharides, citric acid	Sectioned (0.2–0.5 mm) at room temperature with knife. Fresh samples mounted to steel surface without use of adhesive	Li et al. (2007)	HPLC
2007	Wheat (*Triticum aestivum*)	Seed	MALDI, 100 µm	Metabolites, amino acids, carbohydrates	Cryo-sectioning, CHCA or 9-AA matrix	Burrell et al. (2007)	–
2007	Wheat (*Triticum aestivum*)	Stem	MALDI, 200 µm	Oligosaccharides	Cryo-sectioning (50 µm), CHCA matrix	Robinson et al. (2007)	–
2008	White lily (*Lilium candidum*)	Petal	AP IR-MALDI, 200 µm	GABA, glutamine, saccharides	Mounting of sample directly to stage. No matrix applied	Li et al. (2008)	–
2008	Thales cress (*Arabidopsis thaliana*)	Leaf	MALDI, 200 µm	Glucosinolate	Samples mounted on a MALDI target using a double-sided adhesive tape with the abaxial surface of the leaf facing up. 9-AA matrix	Shroff et al. (2008)	HPLC
2009	Sunflower (*Helianthus annuus*)	Stem	MALDI, 200 µm	Nicosulfuron (pesticide)	Cryo-sectioning, CHCA matrix	Anderson et al. (2009)	–
2009	Peach (*Prunus persica*)	Fruit	MALDI, 400 µm	Lipid transfer protein	Cryo-sectioning (250 µm), thaw mounted onto ITO slides, sinapinic acid matrix	Cavatorta et al. (2009)	HPLC–ESI–MS
2010	Rice (*Oryza sativ*a)	Seed	MALDI, 100 µm	Lipids and other metabolites	Cryo-sectioning (8 µm), DHB matrix	Zaima et al. (2010)	MS/MS
2010	Juvenile poplar (*Populus deltoids*)	Stem	MALDI, 20 µm	Cellulose compounds	Cryo-sectioning (50 µm), DHB matrix	Jung et al. (2010)	–
2010	Thale cress (*Arabidopsis thaliana*); date palm *(Phoenix* sp.)	Leaf	MALDI, 200 µm	Cuticular lipids	Mounting of samples using double-sided tape, DHB matrix	Vrkoslav et al. (2010)	GS-MS
2010	Eggplant (*Solanum melongena*)	Fruit	MALDI, 200 and 25 µm	GABA, amino acids, carbohydrates	Cryo-sectioning (14 µm), DHB matrix	Goto-Inoue et al. (2010)	MS/MS
2010	Thale cress (*Arabidopsis thaliana*)	Petal	MALDI/LDI 10–40 µm	Flavonol glycosides		Perdian and Lee (2010)	FT MS, LIT MS, MS/MS, and MS3
2011	*Phyllanthus urinaria*	Leaf	MALDI	Photolabile metabolites	Samples fixed on sample plates using thin layer of resin and kept in vacuum for 10 min before measurement. DHB matrix	Hsiao et al. (2011)	–
2011	Eastern cottonwood (*Populus deltoides*)	Stem	MALDI, 50 µm	Cellulose	Sectioning on vibratome (50 µm), DHB matrix	Lunsford et al. (2011)	MS/MS
2012	Petunia (*Petunia* × hybrid)	Leaf	MALDI, 100 µm	Cyclotides	Cryo-sectioning (15 µm), CHCA matrix	Poth et al. (2012)	LC–MS/MS
2012	Rabbiteye blueberry (*Vaccinium ashei*)	Fruit	MALDI, 100 µm	Anthocyanin’s	Cryo-sectioning (50 µm), DHB matrix	Yoshimura et al. (2012a)	MS/MS
2012	Barley (*Hordeum vulgare*); tobacco (*Nicotiana tabacum*)	Grain, root	MALDI, 15–35 µm	Lipids	Cryo-sectioning (20–55 µm), vacuum dried, DHB and HCCA matrix	Peukert et al. (2012)	MS/MS
2012	Potato (*Solanum tuberosum*)	Tuber	MALDI, 200 µm	Glycoalkaloids	Cryo-sectioning (6 µm), DHB matrix	Ha et al. (2012)	–
2012	Capsicum (*Capsicum annuum*)	Fruit	MALDI, 250 µm	Capsaicin	Cryo-sectioning (70 µm), CHCA matrix	Taira et al. (2012)	–
2012	Cotton (*Gossypium hirsutum*)	Embryos of cotton	MALDI, 50 µm	Lipids	Lightly fixed with paraformaldehyde, cryo-sectioned (30 µm) then mounted on glass slides, DHB matrix	Horn et al. (2012)	
2012	Thale cress (*Arabidopsis thalliana*)	Flower bud, sepal, silique	MALDI, 50 µm	Glucosinolates	Mounted using conductive tape, 9-AA matrix	Sarsby et al. (2012)	–
2012	Black rice (*Oryza sativa*)	Seed	MALDI, 50 µm	Anthocyanin’s, lipids	Embedding in 2 % CMC and cryo-sectioning (10 µm), DHB matrix	Yoshimura et al. (2012b)	MS/MS, HPLC
2012	Apple (*Malus domestica*)	Fruit	MALDI, 75–150 µm	Glycosylated flavonols and dihydrochalcones	Manual slicing with razor blade, CHCA matrix	Franceschi et al. (2012)	–
2013	*Medicago truncatula*–*Sinorhizobium meliloti* symbiosis	Nodulated roots	MALDI	Metabolites during N-fixation	Excised nodules were gelatin embedded and flash-frozen, cryo-sectioned (12 µm) and thaw-mounted on a MALDI plate or ITO-coated glass slides then dehydrated, DHB and DMAN matrices	Ye et al. (2013)	
2013	*Camelina sativa*	Seeds	MALDI, 25 µm	Membrane and storage lipids	Gelatin embedded desiccated seeds were cryo-sectioned (30–50 µm), freeze-dried onto glass slides, DHB matrix	Horn et al. (2013b)	
2013	Hybrid poplar (*Populus tremula* × *Populus alba*), *Rosa hybrida* cv., *Petunia hybrida* cv.	Leaves	MALDI, 50 μm	2-phenylethanol	Fresh leaves of transgenic poplars securely placed on a MALDI target with double-sided tape, the dried in a vacuum chamber, DHB and CHCA as matrices	Costa et al. (2013)	
2013	*Populus nigra, Ambrosia trifida, Artemisia absinthium,* and *Hibiscus syriacus*	Pollen grains	MALDI, 50–150 µm	Metabolites	Grains were fixed onto ITO-coated glass slides	Weidner et al. (2013)	
2013	Avocado (*Persea americana*)	Mesocarp tissue	MALDI	Lipid droplets	Tissue print generated on nitrocellulose membrane, adhered to a stainless-steel slide with double-sided tape, DHB matrix	Horn et al. (2013a)	
2014	Licorice (*Glycyrrhiza glabra*)	Rhizome	AP-SMALDI 10–30 µm	Saponins	Cryo-sectioned (20 µm), thaw mounted on regular glass slides, vacuum dried, DHB matrix	Li et al. (2014a)	
2014	Grape vine (*Vitis vinifera*)	Fruit	AP-SMALDI, 10 µm	Amino acids, carbohydrates and anthocyanin’s	Cryo-sectioned (60 µm), mounted on glass slides, DHB matrix.	Berisha et al. (2014)	
2014	Podophyllum species	Rhizome	MALDI	Alkaloids	Ultra-pure agarose embedded and cryo-sectioned (15 µm), DHB matrix	Marques et al. (2014)	
2014	Tomato (*Solanum lycopersicum*), nectarine (*Prunus persica*) and apple (*Malus domestica*)	Cutins	MALDI	Hydrolyzed cutin and suberin polymers	Mounted on ITO-coated glass slides using conductive carbon tape, in situ alkaline degradation of cutin and suberin polymers, Lithium-doped DHB matrix	Velickovic et al. (2014)	
2014	Radish (*Raphanus sativus*)	Bulbs and leaves	MALDI 150 µm, MALDI, SIMS	N-labelled choline and phosphocholine	Bulbs were snap-frozen, cryo-sectioned (12 µm), mounted on ITO-coated glass slides. Leaves were freeze dried between two glass slides, mounted on aluminum or glass slides with double-sided carbon tape, CHCA and DHB matrix	Seaman et al. (2014)	MS/MS
2014	Wheat (*Triticum aestivum* L)	Grain	MALDI, 100 µm	Cell-wall polysaccharides: acetylated arabinoxylan, beta glucans	Embryo was excised and the grain sectioned with a vibratome (60 µm), sections washed with 50 % EtOH, mounted on ITO-coated glass slides, in situ digestion of cell-wall polysaccharides, DHB-DMA and aniline-DHB matrix	Veličković et al. (2014)	
2014	Tomato (*Solanum lycopersicum*)	*Bacillus amyloliquefaciens* S499 infected seedlings roots	MALDI, 150 µm	S499 antibiome: lipopeptide (LP)	Poured onto ITO glass slides, vacuum dried, CHCA matrix	Debois et al. (2014)	
2014	Barley (*Hordeum vulgare*)	Grain	MALDI, 15–30 µm	Hexoses, sucrose, fructans	OCT fixed and cryo-sectioned (30 µm), mounted on ITO-coated glass slides, DHB matrix	Peukert et al. (2014)	
2014	Maize (*Zea mays*)	Seedling leaf	MALDI, 25 µm	Small molecules	Gelatin embedded, cryo-sectioned (10 µm), 9-AA and DAN matrices	Korte and Lee (2014)	
2014	Vine tomato (*Solanum lycopersicum*)	Fruit	MALDI, 250 µm	Lipid transfer proteins	CMC-embedded and cryo-sectioned (50 µm), mounted on ITO slides, CHCA-Aniline matrix	Bencivenni et al. (2014)	
2014	*Medicago truncatula*	Root nodules	MALDI, 50 µm	Small molecules: organic acids, amino acids	Gelatin-frozen nodules, cryo-sectioned (8–20 µm) and thaw mounted on ITO glass slide, DHB matrix.	Gemperline and Li (2014a)	
2014	Eucalyptus	Seedlings stem	MALDI, 50 μm	Lignin monomers and oligomers	Manually sectioned (∼1.5 mm thick) with a sharp razor blade, fixed on glass slides using double-sided tape, silica TLC powder as matrix	Araújo et al. (2014)	
2014	Grapevine (*Vitis vinifera)*	Leaf	MALDI, 50 μm	Resveratrol, pterostilbene and viniferins	Leaf discs, fixed on metal MALDI target with aluminized tape, DAN, CHCA, THAP, 9AA and TFA acidified: DHB, CHCA and THAP matrices	Becker et al. (2014)	
2014	Cotton (*Gossypium hirsutum*)	Seeds	MALDI, 50–75 µm	TAGs	Gelatin embedded mature embryos, cryo-sectioned and freeze-dried on glass slides, DHB matrix	Horn et al. (2014)	
2015	Citrus (*Citrus sinensis* and *Citrus limonia*)	Leaf, Stem	MALDI, 35 μm	Hesperidin and rutin	Samples sectioned using microtome (20 μm) mounted with double-sided tape to ITO-coated glass slides, CHCA and DHB matrices	Soares et al. (2015)	MALDI-TOF/TOF, HPLC–UV
2015	Maize (*Zea mays*)	Leaf	MALDI, 5 μm	Amino acids, glycerolipids, and defense-related compounds	Gelatin embedded and rapidly frozen with liquid nitrogen, transverse cryo-sections (10 μm), DAN matrix applied via sublimation	Korte et al. (2015)	MS/MS performed using ion trap analyzer
2015	Thale cress (*Arabidopsis thaliana*)	Leaf	MALDI, 50 μm	Glucosinolates	Samples mounted to glass slides with double-sided adhesive tapes, 9-AA matrix applied via sublimation	Shroff et al. (2015)	MS/MS, LAESI-QTOF and LESA using ESI-HDMS and ESI-Orbitrap
*Secondary Ion Mass Spectrometry*							
2005	Sugi tree (*Cryptomeria japonica*)	Wood tissue	SIMS 15 keV Ga + beam, resolution unknown	Ferruginol	Tissue sectioning (30 µm) of heartwood and sapwood prepared using a microtome, samples attached to silicon plates and covered with stainless steel mesh	Imai et al. (2005)	GC–MS
2008	Hinoki cypress (*Chamaecyparis obtuse*)	Wood tissue	SIMS 2 µm spot diameter, 22 keV Au1 + beam	Hinokiresinol, hinokione, hinokiol, hinokinin	Microtome sectioning (100 µm), dried at room temperature	Saito et al. (2008)	–
2010	Peas (*Pisum sativum*) and thale cress (*Arabidopsis thaliana*)	Seed	SIMS 25 keV Bi3 + beam	Flavonoid	Pea seeds were cryo-sectioned (12 µm) then deposited onto silicon wafers, dried under vacuum for 15 min, without any further treatment. Arabidopsis seeds prepared according to established sample preparation procedures for histology/scanning electron microscopy	Seyer et al. (2010)	–
2011	Rice (*Oryza sativa*)	Roots	Nano-SIMS 100 nm, 16-keV Cs + ion beam	Silicon, arsenic	Rice roots sectioned under MES buffer using a scalpel blade, sections placed into planchettes, freeze-substituted and embedded in low viscosity resin, 1 µm sections for nano-SIMS	Moore et al. (2011)	
2011	Poplar (*Populus trichocarpa*)	Wood tissue	SIMS 300 nm spot diameter 25 keV Bi3 + beam	Guaiacyl and syringyl lignin units	Dehydration, incubation in wax, microtome sectioning, incubation in wax, dewaxing and drying	Zhou et al. (2011)	–
2012	Maple (*Acer. micranthum*)	Wood tissue	SIMS 1–2 µm spot diameter, 22 keV Au1 + beam	Guaiacyl and syringyl lignin units	Microtome sectioning (100 µm)	Saito et al. (2012)	–

Table adapted and extensively extended from Bjarnholt et al. (2014)

**Fig. 5:**
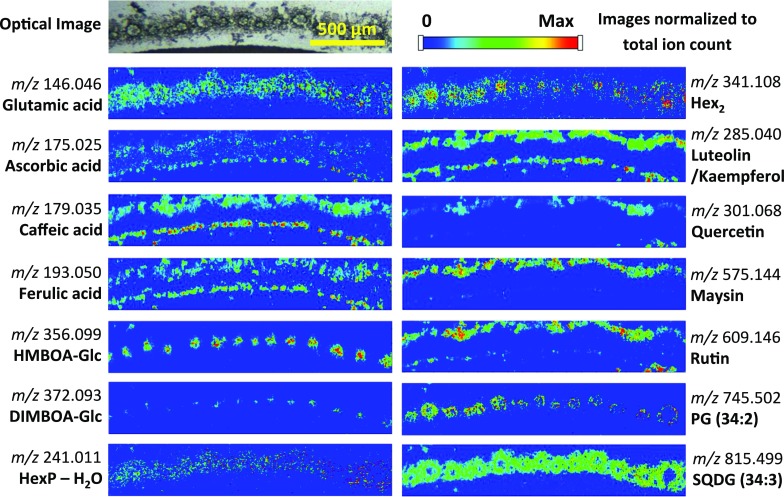
Optical image and MS images of various metabolites in a maize leaf cross-section obtained at 5 µm spatial resolution. Images are oriented such that the upward-facing (adaxial) surface of the leaf is at the top. *HMBOA-Glc* 2-hydroxy-7-methoxy-1,4-benzoxazin-3-one glucoside; *DIMBOA-Glc* 2,4-dihydroxy-7-methoxy-1,4-benzoxazin-3-one glucoside; *HexP* hexose phosphate; *Hex*_*2*_ hexose disaccharide; *PG* phosphatidylglycerol; *SQDG* sulfoquinovosyl diacylglycerol. Glutamic acid and HexP are found throughout the tissues with disaccharides concentrated within the vasculature. Ferulic and caffeic acid are found predominantly within the epidermal layers. Flavonoids are found asymmetrically distributed within the epidermal layers of the tissue. Notably, Maysin is found exclusively in the adaxial epidermis consistent with anti-herbivory and UV protectant properties. PG(34:2) was found exclusively in the bundle sheath cells, SQDG found distributed in bundle sheath and mesophyll cells. HMBOA-Glc and DIMBOA-Glc found to be specifically distributed to select mesophyll cells between the vascular bundles. Signals are normalized to TIC on each pixel. Maximum values for generating images are as follows. Glutamic acid: 1 × 10^−2^. Ascorbic acid: 8 × 10^−3^. Caffeic acid: 3.5 × 10^−2^. Ferulic acid: 8 × 10^−3^. HMBOA-Glc: 3 × 10^−2^. DIMBOA-Glc: 1 × 10^−2^. HexP-H_2_O: 4 × 10^−3^. Hex_2_: 6 × 10^−3^. Luteolin/kaempferol: 5 × 10^−2^. Quercetin: 4.5 × 10^−2^. Maysin: 5 × 10^−2^. Rutin: 2 × 10^−2^. PG (34:2): 5 × 10^−3^. SQDG (34:3): 3 × 10^−2^.Reproduced with kind permission from Springer Science and Business Media, Anal. Bioanal. Chem., (Korte et al., 2015), 407(8):2301–2309, Copyright © 2015

Detection and visualization of primary metabolite distributions using MSI provides insights to normal plant growth, development and reproduction processes. In a multi-omics approach including MALDI-MSI, fructan metabolism of barley grain was studied at different post-pollination time points (Peukert et al. 2014). The authors reported levan- and graminan-type fructan accumulation in the endosperm before starch biosynthesis, while inulin-type fructan was more concentrated in and around the emerging endosperm cavity. Low-weight metabolites, including amino acids, small organic acids, flavonoid and flavonoid glycosides, benzoxazinoids and sulpholipids, have been imaged in corn leaf (Korte and Lee 2014), with cell-level differences in distribution across a wide range of metabolites within juvenile maize leaves determined via an oversampling method in MALDI-MSI at lateral resolutions of 5 µm (Korte et al. 2015) (Fig. 5). The authors found distinct differences in distributions of plant metabolites, including intermediates in central carbon metabolism, cell wall components, lipids and flavonoids. Of note, flavonoids were found asymmetrically distributed within the epidermal layers of the tissue, in particular maysin was present exclusively in the adaxial epidermis, consistent with its reported anti-herbivory (Rector et al. 2003) and UV protectant properties (Casati and Walbot 2005). The plant defense benzoxazinoids, HMBOA-Glc (2-hydroxy-7-methoxy-1,4-benzoxazin-3-one glucoside) and DIMBOA-Glc (2,4-dihydroxy-7-methoxy-1,4-benzoxazin-3-one glucoside) were found to be specifically localized to select mesophyll cells between the vascular bundles. The authors also provide evidence for subcellular distribution of plant metabolites, where they observed non-overlapping localization of DIMBOA-Glc, stored in cell vacuoles, and SQDG in the chloroplast of the same cell.

**Fig. 6:**
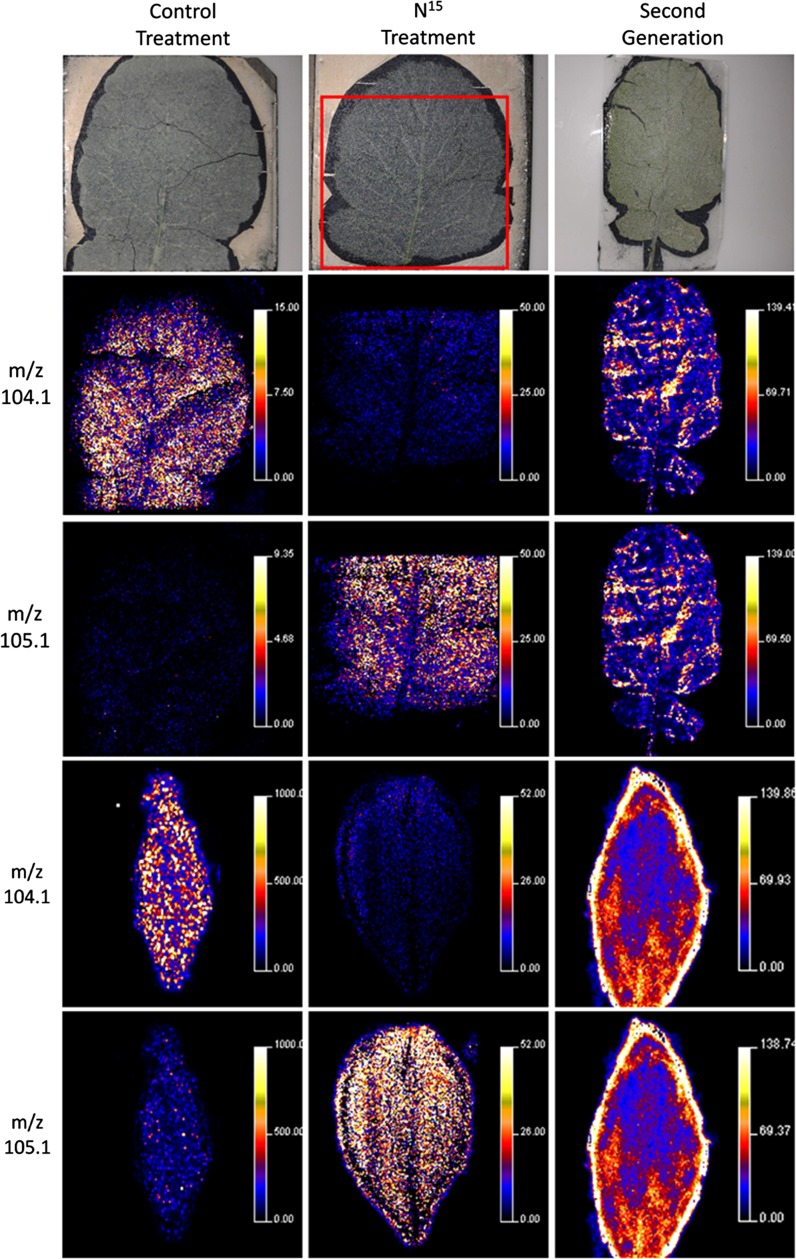
MALDI-MS images showing the distribution of choline at m/z 104 and 105 within the leaf and the bulb of the radish (normalized against TIC) Reprinted with permission from Anal.Chem. (Seaman et al., 2014), 86, 10071–7. Copyright © American Chemical Society

In an impressive exploration of temporal biology, Seaman and colleagues (2014) investigated the nitrogen cycle, from elemental uptake and incorporation into one generation of radish plants, followed by decomposition of the leaves and release of nitrogen, then transfer of labelled nitrogen compounds and incorporation into the next generation (Fig. 6). They monitored labelled ^15^N, which was found to be incorporated into choline and phosphocholine, using both MALDI and SIMS imaging in the so-called ‘Afterlife Experiment’ (Seaman et al. 2014). The experiment demonstrated the usefulness of stable isotope labelling to examine dynamic processes and the recycling of materials from dead to living organisms.

The storage of specialized metabolites in special cell types has been explored by examining the metabolite profiles of trichomes on the leaves of wild tomato, *Solanum habrochaites*. Metabolites were visualized using carbon-substrate-assisted laser desorption/ionization, in which acyl sugars, alkaloids, flavonoids and terpenoid acids were successfully detected at a spatial resolution of around 50 μm (Li et al. 2014b).

### Elemental distributions

Elemental distributions within plant tissues have been investigated using LA-ICP-MS, where the distribution and quantities of the elements selenium and sulphur were mapped in sunflower plant leaves (da Silva and Arruda 2013). Sulphur is incorporated into a variety of primary metabolites but also defense glucosinolates. LA-ICP-MS has also been used to compare the distribution of iron (Fe) concentrations in transgenic and non-transgenic soybean leaves, with differences of spatial distribution identified using LA-ICP-MS. While Fe was homogenously distributed across the whole transgenic soybean leaf, it was concentrated in the leaf mid-vein and secondary veins of non-transgenics (Oliveira and Arruda 2015). The altered distribution of Fe was attributed to the changes in the genome and gene products of the transgenic soy plants. Nanoscale SIMS (NanoSIMS) is a technique used to measure the nanoscale distribution (<100 nm) of elements and isotopes available only on a Cameca NanoSIMS 50 instrument. Using NanoSIMS, the distribution of toxic depositions of arsenic (As) in rice grains and roots has been investigated; As is a severe health threat to rice consumers, and it is important to understand how the rice plant takes up As and distributes it within its tissues (Moore et al. 2013). The authors found a difference of As distribution between high 2-deoxymugineic acid (DMA) grains and wild-type rice grains treated with inorganics. For the former, they found high concentrations in the sub-aleurone region, while for the latter, As was found in the multilayered aleurone layer near the ovular vascular trace (Moore et al. 2013).

### Plant structural components

The composition of plant structural components is of interest to biologists, however most structural components are far too large to measure using MSI approaches. Typically, it is much easier to measure the precursor monomeric units that are directly incorporated into larger structural components. In an example of measuring the spatial distribution of monomeric precursors, syringyl and guaiacyl lignin units have been reported in *Eucalyptus* species, using MALDI-MSI and silica particles as a novel matrix. Examination of differential distribution of monomeric units provides some insight for the purpose of biofuel production from lignocellulosic materials (Araújo et al. 2014). Another strategy to examine the compositional variation in structural units uses enzymatic hydrolysis and tissue pre-treatments to degrade structural components into smaller units which are more amenable to measurement. The localization and quantity of arabinoxylans and beta-glucans in developing wheat grain cell walls was investigated using this approach, where initial in situ enzymatic digestion of large polysaccharides and MALDI-MSI analysis demonstrated an intense endogenous acetylation of arabinoxylans in young grains, as well as feruloylation of arabinoxylans and a variety of structural features of beta-glucans (Veličković et al. 2014). The approach proved effective to measure plant polysaccharide segregation and enabled in situ polysaccharide structural characterization.

### Plant surface metabolites

Mapping of surface-bound metabolites on plant leaves and flowers is another popular area of research, and DESI-MSI provides an exemplary method for directly sampling plant surfaces. It has been used on especially delicate plant surfaces such as flower petals and thin leaves, where it has been extensively used to examine metabolic changes during growth and stress responses (Cabral et al. 2013; Hemalatha and Pradeep 2013; Li et al. 2011, 2013b; Muller et al. 2011). MSI techniques are often hard to apply to non-flat and irregular plant surfaces such as petals, leaves or fruits, because the preparation of the plant sample becomes time-consuming and frequently requires the use of a cryostat. However, such morphological problems may be overcome by using DESI-MSI. This technique allows preserving the relationship between the spatial distribution and the relative intensity of the chemical compounds. Also, the soft tissues of leaves and petals have been examined using a similar DESI approach by employing a ternary solvent system that enabled the direct imaging of Very-Long-Chain Fatty Acids (VLCFAs) and other secondary metabolites in the cuticle (Li et al. 2013a). By employing a ternary solvent system, the cuticle was not removed from the sample and, as a result, VLCFAs were preserved for MSI. Surface heterogeneity of other plant lipid polymers such as cutin and suberin were determined in tomato, apple and nectarine fruits using MALDI-MSI coupled with in situ alkaline depolymerization (Velickovic et al. 2014). This overcame the limitation to analyze structural features of plant surface lipid polymers that would be otherwise difficult to access by dissection and chemical analysis.

### Plant lipids

Lipids, including triacylglycerols (TAGs), glycerophospholipids and sterols, are of particular interest for the generation of high-lipid-content species. MALDI-MSI has been particularly suited to lipid imaging and has been employed in plant-based MSI research. The chemical distribution of the major and minor storage and membrane lipids on mature cotton embryo tissues was examined, and a heterogeneous distribution of TAGs and phosphatidylcholines (PCs) was observed at the cellular level (Horn et al. 2012). MALDI-MSI revealed an altered accumulation of TAG species in cotton embryos expressing a non-functional allele of a *Brassica napus* delta-12 desaturase gene, particularly within cotyledon tissues (Horn et al. 2014). The authors concluded that genetic modifications of cottonseed fatty acid composition are associated with changes in dominant molecular species of TAGs and their spatial distributions within embryo tissues, indicating the possibility of tissue-specific differences in TAG biosynthesis pathways within cotton embryos (Horn et al. 2014). In a truly multi-omics research program combining lipidomics, proteomics and transcriptomics, two new lipid droplet-associated proteins (LDAP1 and LDAP2) were discovered (Horn et al. 2013a). In other species, MALDI-MSI has been employed to map the spatial differences in TAG deposition in lipid bodies present in Avocado mesocarps (Horn et al. 2013a). In an example of multi-modal imaging, they utilized MRI and MSI to examine the distribution of TAGs and their glycerophospholipid precursors within cotyledons and the hypocotyl/radical axis in embryos of *Camelina sativa*, a crop targeted for future biofuel production (Horn et al. 2013b). Both TAGs and glycerophospholipid precursors were distributed differently within the tissues. Transgenic manipulation of seed lipid composition by modification of enzyme expression resulted in altered patterns of distribution of seed storage lipids, thus highlighting the importance of spatial analysis to identify plant biochemical pathways.

### Plant-symbiont systems

Information on the extent of metabolite changes in plant roots is highly valuable, and plant-symbiont associations, such as nitrogen fixation and defense mechanisms, are of significant importance. While untargeted metabolites can be spatially determined in the model legume plant *Medicago truncatula* roots and nodules during nitrogen fixation via MALDI-MSI (Gemperline and Li 2014a), a combination of this method with MS/MS metabolite fragmentation in *Medicago* root nodules and its symbiotic nitrogen-fixing bacteria *Sinorhizobium meliloti* revealed spatial metabolite distributions between *Medicago* roots, nitrogen-fixing root nodules and non-nitrogen-fixing root nodules (Ye et al. 2013). The studies pave the way for understanding the complex relationship between the plant and its symbiont. MSI studies of other plant-symbiont systems also shed light into understanding the distribution of vital compounds involved in these processes within plant roots. Spatio-temporal distribution of bacterial antibiome/antibiotic biofilms on plant roots that confers resistance against phytopathogens were explored using MALDI-MSI (Debois et al. 2013, 2014). In addition, the spatio-temporal distribution of the plant immunity elicitor surfactin was revealed in different quantities and time intervals. Complementary MS/MS was able to identify new variants of plant surfactins that are vital for protection against pathogen infection (Debois et al. 2014).

### Plant responses to abiotic stress

Metabolic changes occur when plants are exposed to external stress, and plants respond in both localized and global manners. MSI has provided a unique tool to explore the spatio-temporal distributions of plant stress metabolites (e.g. phytoalexins, flavonoids etc.) within different cell types. The distribution of glycosylated flavonols and dihydrochalcones in Golden Delicious apples was determined using MALDI-MSI (Franceschi et al. 2012). Glycosides were found to be differentially distributed, with a quercetin-hexoside found in higher abundance directly beneath the cuticle, whereas quercetin-rhamnoside and phloretin-hexoside were found throughout the apple pericarp but with glycosides more concentrated directly underneath the cuticle (Franceschi et al. 2012). The results from this study suggest highly localized, tissue-specific biosynthesis of different flavonoid glycosides. Separately, MSI imaging of transverse sections of *Lychnophora salicifolia* leaves using Tandem MS to distinguish different flavonoids via unique fragmentation patterns revealed a conserved accumulation of the flavonoid vicenin-2 in the top layers of the leaves. Vicenin-2 is believed to protect the plants from extreme sunlight, and the highly specific localization to the top of the epidermis supports this hypothesis (Silva et al. 2014).

### Plant responses to biotic stress

Plants possess a wide range of defense mechanisms that include structural, chemical and protein-based strategies to cope with biotic stresses. Upon exposure to pathogens and/or herbivores, changes in levels of plant defense compounds, mostly secondary metabolites such as alkaloids, hydroxynitrile glucosides, glucosinolates, phenolics and terpenes are observed (Bennett and Wallsgrove 1994). Localization and distribution of a number of these metabolites has been intensively studied using MSI, with an aim to better understand their roles in plants. MSI has been used to monitor changes in glycoalkaloid toxins produced by plants in response to microbial infection using both DESI and MALDI approaches (Cabral et al. 2013; Ha et al. 2012; Tata et al. 2015). Examination of toxins in food products is important both to guarantee food supply but also to examine the underlying biology of plant-pathogen response. The DESI approach was used to examine fluctuation of toxic glycoalkaloids, α-chaconine and α-solanine, in sprouted potatoes infected by the phytopathogen *Pythium ultimum,* at different time intervals and with minimal sample preparation (Tata et al. 2015). Results demonstrate distinct differences in the spatial distribution of specific plant metabolites throughout the tissue and the accumulation of aglycon and glycoalkaloid precursors, including solanidine, solasodine, γ/β-chaconine, γ/β-solanine and others, around the site of infection. At later time points, decreases in these metabolites around the infection demonstrated the ability of the pathogen to metabolize toxic glycoalkaloids to less toxic intermediates, by partial or complete hydrolysis of sugar units. Other glycoalkaloids including saponins have also been examined in *Glycyrrhiza glabra* (licorice) using AP-MALDI-MS/MS at high mass and spatial resolution (10 μm). The results provided unique information, localizing biosynthetic pathways for glycoalkaloid production to the rhizomes, which are the primary source of compounds with medicinal value (Li et al. 2014a). In addition, MALDI-MSI was also able to confirm the presence of the mycotoxin deoxynivalenol on a fungus-infected wheat seed surface (Berisha et al. 2014), as well as illustrate high levels of hesperidin distributed at infection sites of *Xylella fastidiosa* on citrus leaves and stems (Soares et al. 2015). The antifungal secondary metabolites hordatine and its derivatives were also spatially mapped in barley embryo tissues using MALDI-MSI, where specific glycosylation patterns as well as tissue-specific hordatine derivatives were revealed (Gorzolka et al. 2014).

**Fig. 7:**
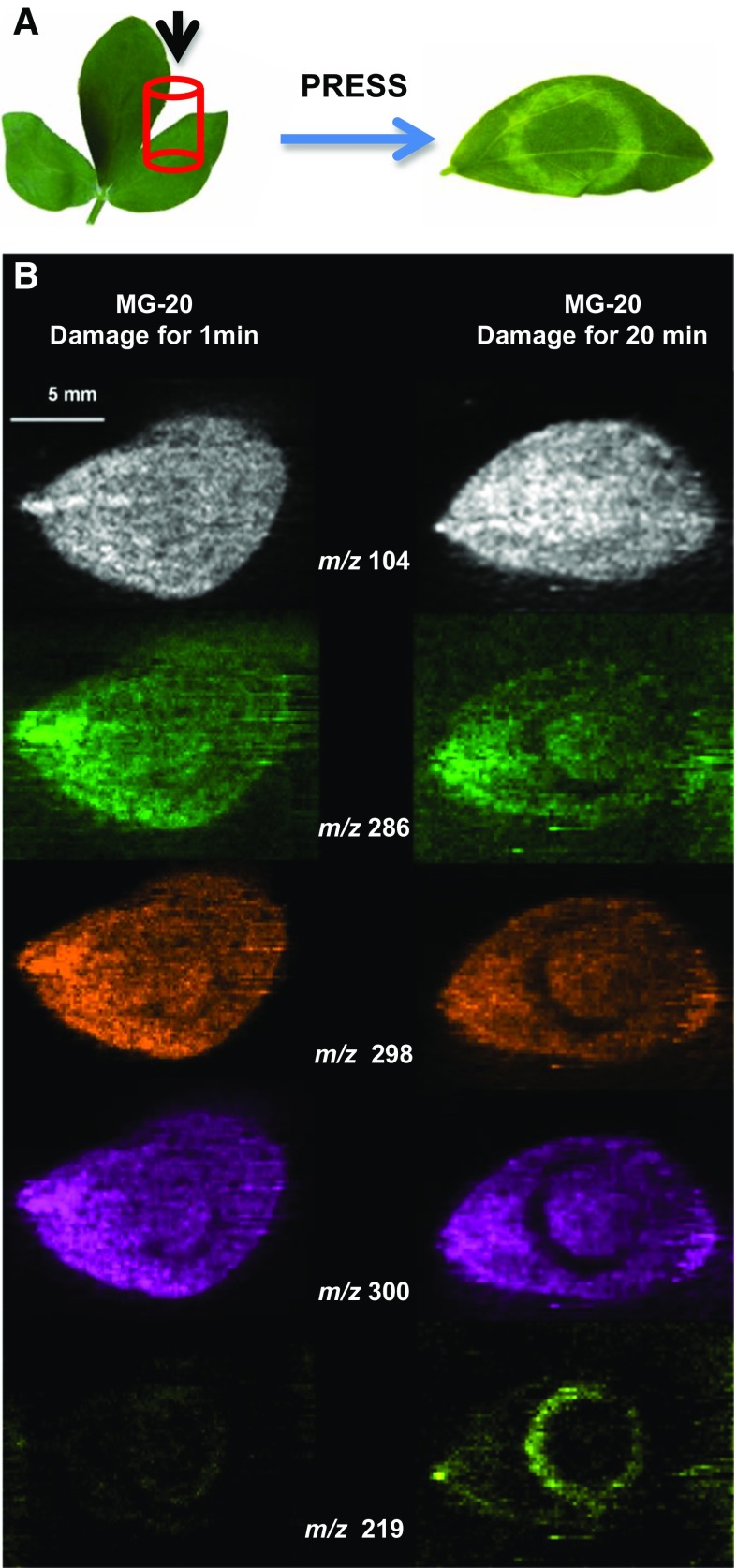
Demonstrates immediate response to physical stress and degradation of hydoxynitriles (cyanogenic glucosides) in wounded *Lotus japonicas* MG20 leaf tissues over time. Visualization of β-glucosidase mediated hydrolysis of hydroxynitrile glucosides in wounded leaves. **A** The leaves were wounded by pressing with a metal pipe; **B** indirect DESI-MS images of the wounded leaves: m/z 104 [γ-aminobutyric acid + H]^+^, 286 [linamarin + K]^+^, 298 [rhodiocyanoside + K]^+^ and 300 [lotaustralin + K]^+^, m/z 219 = [glucose + K]^+^. Reproduced with kind permission from John Wiley and Sons Ltd, The Plant Journal, (Li et al., 2013b), 74:1059-1071, Copyright © 2013

Other plant defense molecules, including the highly toxic hydroxynitrile glucosides (cyanogenic glucosides) have been detected in the plant leaf epidermis using DESI-MSI (Li et al. 2011, 2013b) (Fig. 7). The herbivory response was simulated in *Lotus japonicas* applying mechanical stress to the leaf by crushing. Mechanical stress releases stores of hydroxynitrile glucosides and allows interaction with β-glycosidases and lyases, leading to enzymatic hydrolysis and release of hydrogen cyanide. The reaction is observed as local decreases in hydroxynitrile signal and a corresponding increase of glucose as one of the hydrolysis products in the tissues. MALDI-MSI has been used to determine spatio-temporal distribution and quantities of glucosinolates in *Arabidopsis* leaves, where the glucosinolate profile and overall concentration not only attracts but also affects feeding preferences of lepidopterans (Sarsby et al. 2012; Shroff et al. 2008, 2015). The authors established a robust, quantitative imaging approach to determine the concentrations of glucosinolates on the leaf surface (Shroff et al. 2015). Banana-specific nematostatic and nematicidal phytoalexins, phenylphenalenones, were examined using a multi-modal approach, involving a combination of LDI-MSI, ^1^H NMR spectroscopy and Raman microspectroscopy, to determine the distribution of phenylphenalenones around nematode-caused lesions on banana plants; and their ingestion and localization within nematodes (Hölscher et al. 2014). The results demonstrated that the higher concentration of the phenylphenalenone anigorufone, produced by resistant cultivars, is the reason for differences in cultivar resistance to nematode infection.

## Future outlook

Ongoing technological improvements promise to surpass the limitations of current instruments, for instance where spatially resolved detection using the Timepix detector enables a rapid analysis of larger areas, leading to faster acquisition times for MSI experiments (Soltwisch et al. 2014; Syed et al. 2015). However, the technology is not in common use and is limited to TOF detectors but offers significant promise for profiling- and screening-type MSI approaches where ultra-high mass accuracy is not needed. Continual development of older MSI technologies to bypass current limitations will provide new capabilities; a recent example is SIMS, where development of ‘soft ionization’ techniques using water cluster beams has enabled measurement of the molecular ions of individual lipids without fragmentation to lateral resolutions of less than 10 µm (Berrueta Razo et al. 2014; Sheraz nee Rabbani et al. 2015). The development of new types of sources, including the vast array of ambient pressure sources that could be employed in MSI will reduce necessary sample preparation steps and allow examination of a wider range of surfaces (Monge et al. 2013; Wu et al. 2013). Modification of developed sources, such as addition of a second post-ionization laser to generate the MALDI-2-MS source (Soltwisch et al. 2015), offers much promise to increase the sensitivity towards plant metabolites. New combination approaches that take advantage of complementary technologies on a single instrument provide an enormous advantage where, for example, small molecules may be mapped quickly with MALDI to high lateral resolution, and then metabolite and protein distributions can determined by nano-ESI-LESA (Tomlinson et al. 2014).

**Fig. 8:**
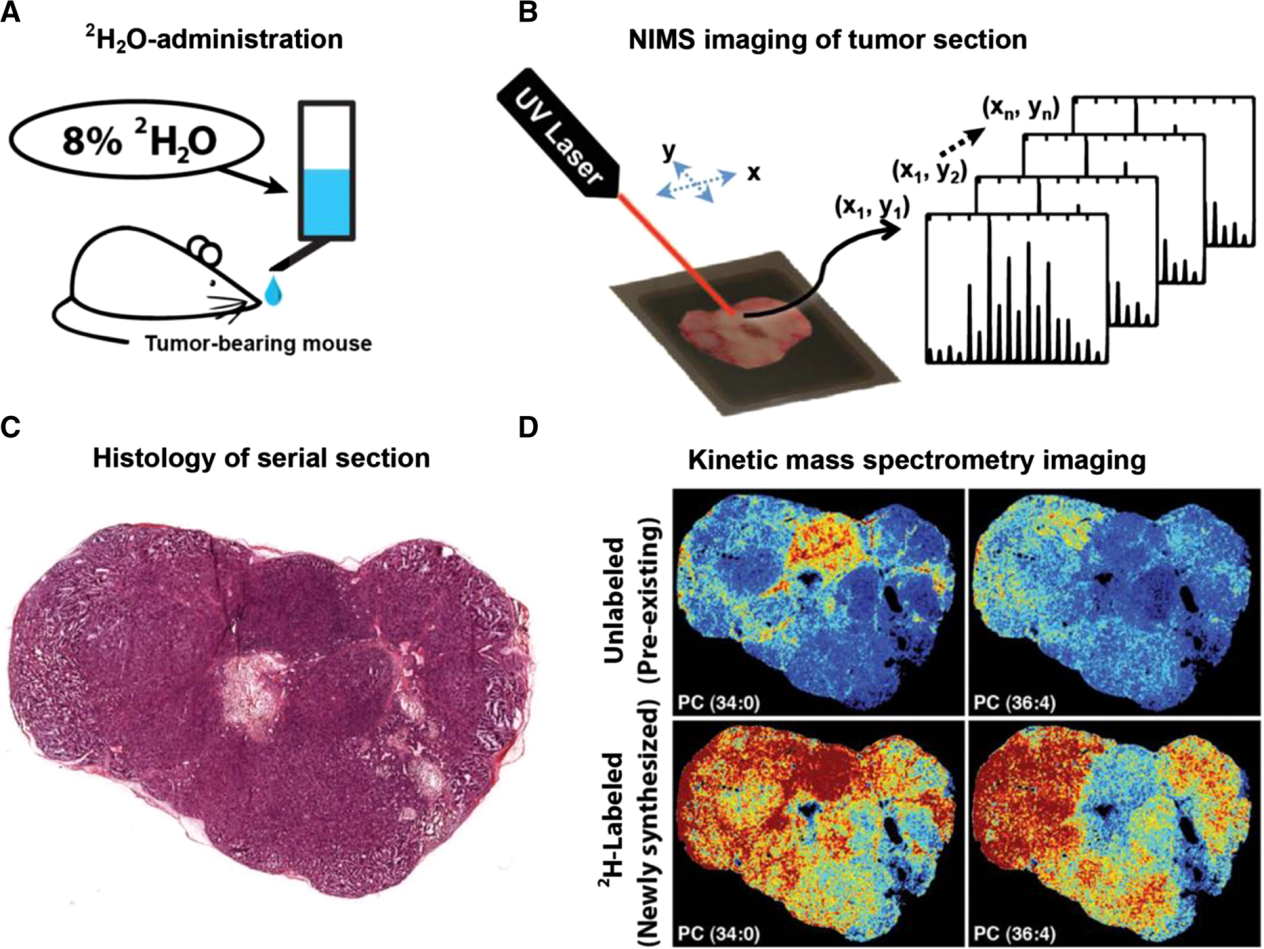
Example of kinetic mass spectrometric imaging—experimental workflow for using kMSI to define spatial heterogeneity of lipid composition and biosynthesis. **A** A tumor-bearing mouse is administered ^2^H_2_O-enriched water to incorporate deuterium into tissue as a result of active metabolism. **B** The deuterium-enriched tumor is excised, sectioned and imaged using NIMS. An individual mass spectrum is generated for each pixel every 50 µm, with spectra comprised of isotopologues from both ^2^H-labeled and unlabeled lipid molecules. **C** Serial sections of the tumor are used for histopathology correlation with kMSI results. **D** Deconvolution of spectra is performed to separate ^2^H-labeled and unlabeled lipids. Intensity images are generated to show the spatial distribution for both newly synthesized and pre-existing lipids. Reprinted by permission from Macmillan Publishers Ltd: Scientific Reports, (Louie et al., 2013) 3:1656, Copyright © 2013

A single MSI experiment provides only a static snapshot of the underlying molecular distribution, which does not allow direct determination of metabolic flux within an organism. Development of new analytical approaches to examine spatio-temporally resolved metabolite flux using Kinetic Mass Spectrometry Imaging (kMSI), through incorporation of stable isotope labelling, provides much potential to explore the dynamics of plant metabolism (Louie et al. 2013) (Fig. 8).

MSI suffers from ion suppression effects and has limitations where molecules cannot be ionized or volatilized. However, the development of ‘Reactive nano-DESI’, which incorporates selective and reactive chemicals (for specific metabolites or classes of metabolites) into the solvent stream, raises new possibilities (Laskin et al. 2012a). Individual classes of chemicals may be targeted, chemically modified to enhance ionization or provide a specific chemical probe that may allow quantification directly off the tissue, providing another level of chemical specificity and a selective tool for the analytical scientist.

Data analysis remains a bottleneck, however, emerging MSI data analysis techniques that enable analysis of ultra-high resolution MSI data and incorporate spatial segmentation will enhance discovery of spatially resolved metabolism. Further development of unsupervised techniques that utilize the spatial information within a MSI dataset and statistical techniques to discover co-occurring metabolites and significant differences in regions of tissue will also help to unlock the power of MSI analysis. Further afield, integration of MSI data with gene expression and metabolomics data will allow identification of novel biosynthetic pathways and mechanisms, providing new avenues to explore biological function of metabolites and genes. Specifically, the integration of the transcriptome with the measured metabolome of an organism has proven to be a powerful method to interrogate production of novel phytochemicals by allowing rapid identification of the genes and gene products involved in underlying biosynthetic pathways (Hegeman 2010; Sumner et al. 2015; Zhang et al. 2004). Plant-based MSI, used as a spatial metabolomics tool, holds much promise to localize the biosynthesis of important plant metabolites and their storage sites, aiding the elucidation of specialized biosynthetic pathways and the identification of genes and gene products. Together, this has the potential to enhance bioengineering of crops to either produce much-needed phytochemicals or to better withstand challenging environmental conditions.

## Conclusion

The MSI research that has been conducted up to this point will underpin the future development of techniques and instrumentation of chemical mapping of plant tissues. Over the past decade, plant MSI has developed rapidly from a boutique technique employed by analytical chemists to a robust technique that is rapidly growing. Plant scientists across a diverse range of research fields are employing MSI to examine fundamental plant biology. Technical developments have overcome many past difficulties, and emerging data analysis methods promise to unleash the full potential of MSI for spatial analysis.

## Abbreviations

3D-MSI: Three-dimensional mass spectrometry imaging
9-AA: 9-Aminoacridine
AP-MALDI: Atmospheric-pressure matrix-assisted laser desorption ionization
AP-SMALDI: Atmospheric-pressure high-resolution scanning-microprobe—MALDI
CHCA (or HCCA): α-Cyano-4-hydroxycinnamic acid
DAN: 1,5-Diaminonaphthalene
*Δm*: Mass difference
DESI: Desorption electrospray ionization
DHB: 2,5-Dihydroxybenzoic acid
DIOS: Desorption ionization on silica
DMAN: 1,8-Bis(dimethylamino)naphthalene
ESI: Electrospray ionization
FFPE: Formalin-fixed paraffin-embedded
fNPs: Functional iron nanoparticles
FT: Fourier transform
FT-ICR: Fourier transform ion cyclotron resonance
FT-IR: Fourier transform infrared
FWHM: Full width at half maximum
HCA: Hierarchical cluster analysis
ICP: Inductively coupled plasma
IR-MALDI: Infrared matrix-assisted laser desorption ionization
ITO: Indium tin oxide
kMSI: Kinetic mass spectrometry imaging
LA-ICP: Laser ablation inductively coupled plasma
LAESI: Laser ablation electrospray ionization
LDI: Laser desorption ionization
LESA: Liquid extraction surface analysis
LTP: Low temperature plasma
*m*: Nominal mass
MALDI: Matrix-assisted laser desorption ionization
MRI: Magnetic resonance imaging
MS: Mass spectrometry
MS^n^: Multistage tandem mass spectrometry
MSI: Mass spectrometry imaging
MS/MS: Tandem mass spectrometry
*m*/*z*: Mass-to-charge ratio
nanoDESI: Nanospray desorption electrospray ionization
nano-LC-ESI: Nanoliter liquid chromatography electrospray ionization
Nd:YAG: Neodynium-doped yttrium aluminium garnet
NIMS: Nanostructure initiator mass spectrometry
OCT: Optimal-cutting temperature
PC: Phosphatidylcholine
PCA: Principal component analysis
PTFE: Polytetrafluoroethylene
ROI: Region of interest
RP: Resolving power
SIMS: Secondary ion mass spectrometry
TAG: Triacylglycerol
Tandem MS/MS: Tandem mass spectrometry
TOF: Time-of-flight
TOF-SIMS: Time-of-flight secondary ion mass spectrometry
UV-LDI: Ultraviolet laser desorption ionization
VLCFA: Very-long-chain fatty acid
